# GLP-1 and GIP agonism has no direct actions in human hepatocytes or hepatic stellate cells

**DOI:** 10.1007/s00018-024-05507-6

**Published:** 2024-11-28

**Authors:** Natália da Silva Lima, Alba Cabaleiro, Eva Novoa, Cristina Riobello, Patrick J. Knerr, Yantao He, Eva M. Esquinas-Román, Ismael González-García, Vincent Prevot, Markus Schwaninger, Carlos Dieguez, Miguel López, Timo D. Müller, Marta Varela-Rey, Jonathan D. Douros, Ruben Nogueiras

**Affiliations:** 1 Department of Physiology, CIMUS, University of Santiago de Compostela, 15782 Santiago de Compostela, Spain; 2 CIBER Fisiopatologia de la Obesidad y Nutrición (CIBERobn), Madrid, Spain; 3 Department of Biochemistry and Molecular Biology, CIMUS, University of Santiago de Compostela, 15782 Santiago de Compostela, Spain; 4 Indiana Biosciences Research Institute, Indianapolis, IN USA; 5 Novo Nordisk Research Center Indianapolis, Indianapolis, IN USA; 6 Laboratory of Development and Plasticity of the Neuroendocrine Brain, Lille Neuroscience & Cognition, UMR-S 1172, Univ. Lille, Inserm, CHU Lille, European Genomic Institute for Diabetes (EGID), 59000 Lille, France; 7 Institute for Experimental and Clinical Pharmacology and Toxicology, University of Lübeck, Lübeck, Germany; 8 Institute for Diabetes and Obesity, Helmholtz Diabetes Center, Helmholtz Munich, Neuherberg, Germany; 9 German Center for Diabetes Research (DZD), Neuherberg, Germany; 10 Walther-Straub Institute of Pharmacology and Toxicology, LMU Munich, Munich, Germany; 11 Galician Agency of Innovation (GAIN), Xunta de Galicia, Santiago de Compostela, Spain

**Keywords:** Fatty acids, Liver fibrosis, GLP-1R, GIPR

## Abstract

The use of incretin agonists for managing metabolic dysfunction-associated steatohepatitis (MASH) is currently experiencing considerable interest. However, whether these compounds have a direct action on MASH is still under debate. This study aims to investigate whether GLP-1R/GIPR agonists act directly in hepatocytes and hepatic stellate cells (HSCs). For this, human hepatocyte and HSCs lines, as well as primary human hepatocytes and HSCs treated with Liraglutide, Acyl-GIP or the GLP-1/GIP dual agonist (MAR709) were used. We show that the concentrations of each compound, which were effective in insulin release, did not induce discernible alterations in either hepatocytes or HSCs. In hepatocytes displaying elevated fatty acid content after the treatment with oleic acid and palmitic acid, none of the three compounds reduced lipid concentration. Similarly, in HSCs activated with transforming growth factor-β (TGFb), Liraglutide, Acyl-GIP and MAR709 failed to ameliorate the elevated expression of fibrotic markers. The three compounds were also ineffective in phosphorylating CREB, which mediates insulinotropic actions, in both hepatocytes and HSCs. These findings indicate that incretin agonists have no direct actions in human hepatocytes or hepatic stellate cells, suggesting that their beneficial effects in patients with MASH are likely mediated indirectly, potentially through improvements in body weight, insulin resistance and glycemic control.

**Supplementary Information:**

The online version contains supplementary material available at 10.1007/s00018-024-05507-6.

## Introduction

With an estimated worldwide prevalence of at least 25%, metabolic dysfunction-associated steatohepatitis (MASH) is a prominent cause of end-stage liver disease and a primary reason for liver transplantation globally. The increasing incidence of MASH correlates strongly with the rising rates of obesity and type 2 diabetes (T2D), plus the impacts of the environment, the microbiome, comorbidities, and genetic predisposition. Despite the extensive research and numerous clinical trials exploring potential drug candidates, only resmetirom has been approved by the FDA for treatment of MASH with advanced fibrosis [9]. Weight loss and improvement of insulin sensitivity are cornerstones of MASH therapy [39, 46], albeit doing so may not be sufficient by itself to attenuate MASH progression [11]. For instance, bariatric surgery has demonstrated significant long-term benefits for MASH patients, with notable improvements in histological MASH and fibrosis observed five years post-operation [24]. However, the invasive nature, associated risks, and high costs of bariatric surgery limit its accessibility to the majority of MASH patients.

Glucagon-like peptide-1 (GLP-1) and glucose-dependent insulinotropic polypeptide (GIP) are incretin hormones secreted by the gastrointestinal tract in response to food intake. These hormones exert multiple physiological effects, including the stimulation of insulin secretion, reduction of food intake, decelerations of gastric emptying and enhancement of insulin sensitivity [1, 35, 50]. As several GLP-1 receptor agonists have been licensed for the treatment of diabetes and obesity, they were also evaluated in patients with MASH, and successful phase 2a and 2b studies have resulted in progression to phase 3 clinical trials [37]. Recent evidence suggests that dual agonism of GIP/GLP-1 may offer superior efficacy compared to placebo in resolving MASH [12, 26].

Despite the promising results regarding the efficacy of a unimolecular GLP-1/GIP dual agonist in improving liver function, there is an ongoing debate on whether GLP-1R and GIPR agonists have direct effects on MASH or whether they impact on pathophysiology through improvements in weight, insulin resistance and glycemic control. The controversy is due to some studies reporting the expression of GLP-1R in human hepatocytes [49] and suggesting a role in decreasing hepatic steatosis [16, 43], while others failed to detect hepatic expression of GLP-1R [22, 23].

The aim of the current work is to investigate the effects of a long-acting acylated (Acyl) GLP-1 analog (Liraglutide), Acyl-GIP and a GLP-1/GIP dual agonist MAR709 directly in primary human hepatocytes and hepatic stellate cells (HSCs) as well as in cell lines for both cellular types. The Acyl-GIP analog used in this study was previously shown to decrease LDL cholesterol and atherosclerosis in LDLR KO mice [41], and to reduce body weight and food intake via central GIPR agonism [25, 34, 50], without affecting energy and glucose metabolism in mice deficient for *Glp1r* [34, 50]. Our findings indicate that neither individual nor dual agonism of GLP1R/GIPR exerts direct actions on hepatocytes or HSCs. Treatment with these compounds did not ameliorate fatty acid overload in primary human hepatocytes or a human hepatocyte cell line. Similarly, the activation of human HSCs, characterized by a transition from a quiescent state to a proliferative, migratory, and fibrogenic phenotype that is characteristic of liver fibrogenesis, remained unaffected by these drugs.

## Results

### Receptors of GLP-1 and GIP are not expressed in the liver of people with MASLD/MASH

First, we checked the expression of both *GLP1R* and *GIPR* in a public dataset (GEO accessions GSE135251) consisting of bulk RNA sequencing results from samples of patients with MASH at various fibrosis stages and healthy liver controls. *GLP1R* expression is nearly zero (Supplementary Fig. 1A) and *GIPR* expression is very low (Supplementary Fig. 1B). In fact, genes with a read count of less than 10 are often considered artefacts or "noise" in downstream analysis. To elucidate the expression of both receptors in specific hepatic cell populations, we next examined the Human Liver Cell Atlas (https://www.livercellatlas.org/umap-humanAll.php), and the result showed that *GLP1R*, and *GIPR* are hardly expressed in hepatic cells. *GLP1R* was only detected in 17 cells (< 0.1%) in isolated endothelial, stromal, circulating NK/NKT cells and in hepatocytes (Supplementary Fig. 1C). *GIPR* was only found in 0.8% of the total cells, being cholangiocytes and cDC2s the most positive clusters, while a more residual expression was visualized in stromal, hepatocytes, basophils, NK, B and cDC1 cells (Supplementary Fig. 1D). Finally, we examined a public domain dataset to find evidence of *GLP1R* and *GIPR* expression in human liver. To this aim, we analyzed a recent single cell RNA-seq dataset including livers from healthy and cirrhotic individuals (GRO accessions: GSE185477, GSE174748 and GSE212046) [3, 10]. In this case, the *GLP1R* and *GIPR* genes were not present in the merge dataset, which contains only the intersection of the genes expressed in the 3 datasets after QC filtering. Looking at the expression of both genes in the individual datasets, we observed that in the case of *GLP1R*, there is only slight expression (0.15% of the total cells) in the GSE174748 dataset, while GIPR is present in 1.42% of the total cells in GSE174748 and in 0.75% of the cells in GSE212046. In the case of *GLP1R*, the positive cells are almost exclusively hepatocytes (Supplementary Fig. 1E), whereas in the case of *GIPR* there is a consistently higher presence of positive cells in the hepatocytes, cholangiocytes, CD4 T cells and plasma cells clusters in both datasets (Supplementary Fig. 1F, G).

### GLP-1 or GIP agonism does not alter lipid content in human hepatocytes

Despite the gene expression data suggest that incretin receptors are not expressed at meaningful levels in liver cells, we wanted to ensure whether these agonists could work through signaling pathways other than the canonical receptor such as non-specific binding to other G-protein coupled receptors. For this, we explored the possibility that the beneficial actions of GLP-1/GIP dual agonism in the liver of animals [28] and patients [12, 17] with MASH could be exerted through actions within two of the most relevant cell populations involved in the progression of liver fibrosis: hepatocytes and HSCs. Hepatocytes are the most abundant cells in the liver and the relationship between hepatocytes and MASH is central to the understanding of the disease [44], while activation of HSCs is the cellular source of matrix protein-secreting myofibroblasts, the major driver of liver fibrogenesis [18]. To note, we used LX2 human hepatic stellate cells and THLE2 human hepatocyte cells. In agreement with human databases, the staining of GLP-1R in these human cell lines was undetectable compared to INS-1 insulinoma cell line, which showed clear staining (Supplementary Fig. 2A). The staining of GIPR was not assessed since we could not find a specific antibody. Moreover, the gene expression of *GLP1* and *GIPR* was very low compared to subcutaneous white adipose tissue (Supplementary Fig. 2B, C).

**Fig. 1:**
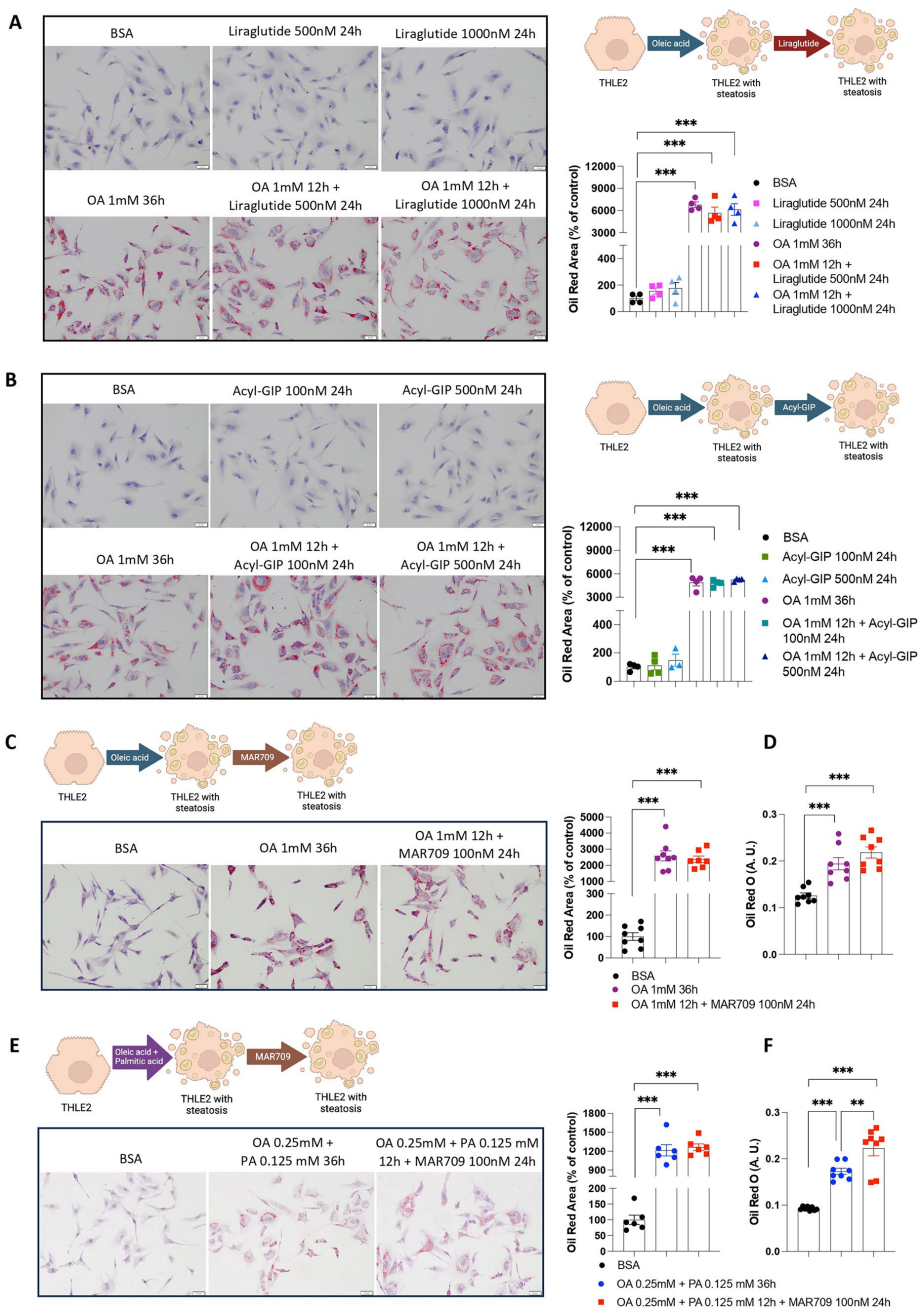
Effects of Liraglutide, Acyl-GIP and GIP/GLP-1 dual agonist in THLE2 human hepatocytes cells on lipid droplet accumulation. **A** Oil Red O staining in THLE2 cells treated with or without oleic acid (OA) 1 mM (12 h) and with Liraglutide (500 nM and 1000 nM, 24 h) (*n* = 4). **B** Oil Red O staining in THLE2 cells treated with or without OA (1 mM, 12 h) and with Acyl-GIP (100 nM and 500 nM, 24 h) (*n* = 4). **C**, **D** Oil Red O staining and Oil Red O quantification by spectrophotometry in THLE2 cells treated with or without OA (1 mM, 12 h) and with MAR709 (100 nM, 24 h) (*n* = 8). **E**, **F** Oil Red O staining and Oil Red O quantification by spectrophotometry in THLE2 cells treated with or without OA (0.25 mM) and palmitate (PA) (0.125 mM) for 12 h and with MAR709 (100 nM, 24 h) (*n* = 6 and *n* = 8, respectively). Data are presented as mean ± SEM; **p < 0.01, ***p < 0.001, using a one-way ANOVA followed by a Bonferroni’s multiple comparison test

To set up functional concentrations of each compound, we used INS-1 cells, which is a well-established model for insulin secretion regulation and pancreatic islet beta-cell function studies. We treated them with Liraglutide (500 and 1000 nM), Acyl-GIP (100 and 500 nM) and MAR709 (50, 100 and 500 nM) [25]. At all the tested concentrations, the compounds stimulated insulin levels as detected by immunofluorescence and with an ELISA kit (Supplementary Fig. 3A–C). Therefore, we treated THLE2 -a human hepatocyte cell line- with oleic acid (OA) or OA with Liraglutide and found that the OA-induced lipid content remained unaltered after the incubation with Liraglutide at the two tested concentrations, as detected by oil red O staining (Fig. 1A). Similar results were obtained when the cells were treated with Acyl-GIP (Fig. 1B) or MAR709 (Fig. 1C, D and Supplementary Fig. 4A), which did not modify the high lipid concentration caused by OA. We also tested a combination of OA with palmitic acid, which as expected augmented intracellular fat content, and again, MAR709 did not produce any effect on lipid content, as measured by both oil red O staining and spectrophotometry (Fig. 1E, F and Supplementary Fig. 4B).

**Fig. 2:**
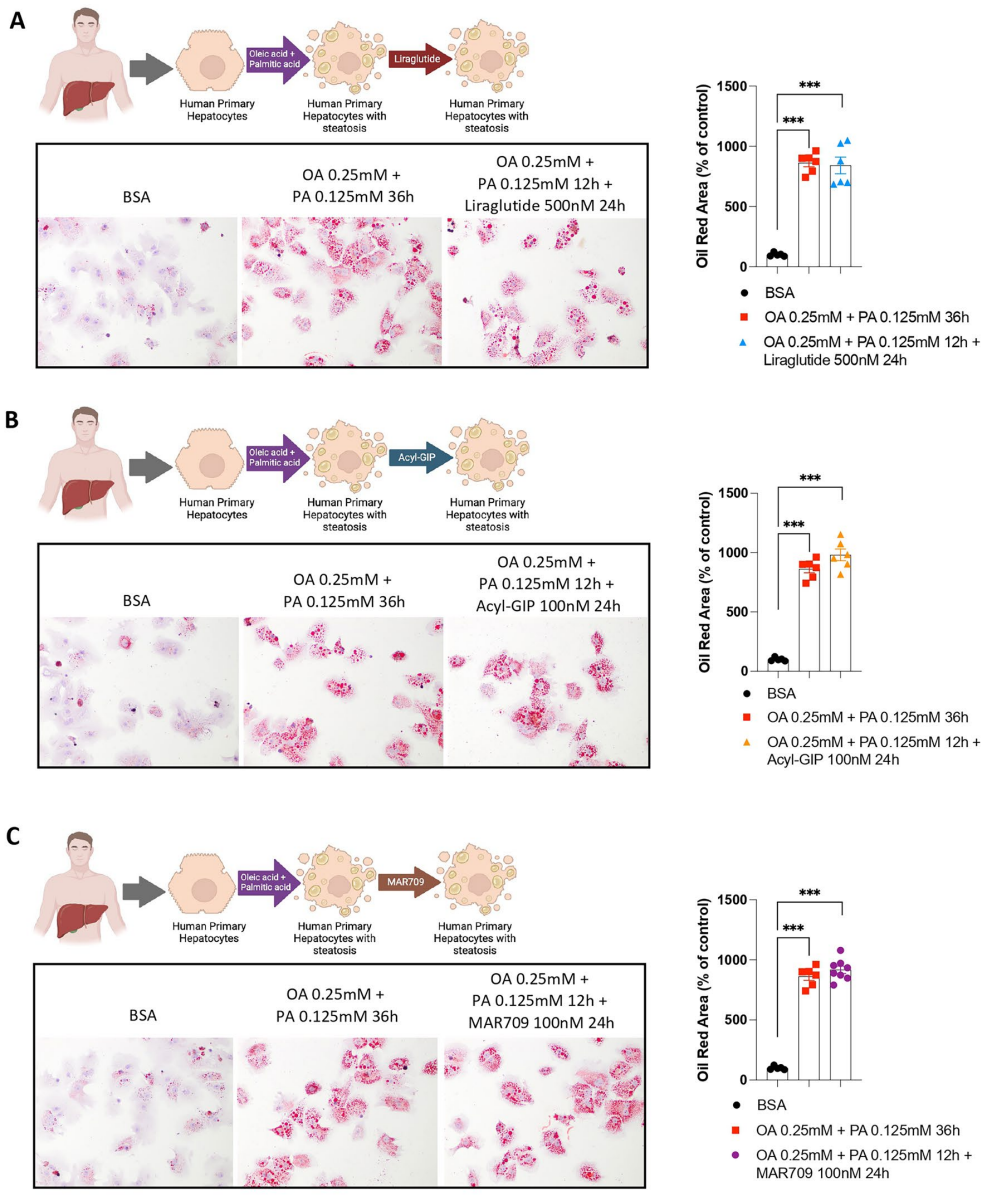
Effects of Liraglutide, Acyl-GIP and GIP/GLP-1 dual agonist in primary human hepatocytes on lipid droplet accumulation. **A** Oil Red O staining in primary human hepatocytes treated with or without oleic acid (OA) (0.25 mM) and Palmitate (PA) (0.125 mM, 12 h) and with Liraglutide (500 nM, 24 h) (*n* = 6). **B** Oil Red O staining in primary human hepatocytes treated with or without OA (0.25 mM) and PA (0.125 mM, 12 h) and with Acyl-GIP (100 nM, 24 h) (*n* = 6). **C** Oil Red O staining in human primary hepatocytes cells treated with or without OA (0.25 mM) and PA (0.125 mM, 12 h) and with MAR709 (100 nM, 24 h) (*n* = 8). Data are presented as mean ± SEM; ***p < 0.001, using a one-way ANOVA followed by a Bonferroni’s multiple comparison test

We next performed experiments in primary human hepatocytes. More specifically, primary hepatocytes were treated with the combination of OA with palmitic acid and then with Liraglutide (Fig. 2A), Acyl-GIP (Fig. 2B) or MAR709 (Fig. 2C). Neither the treatment with the individual incretins nor the dual-agonist were able to reverse the increased fat content caused by the combination of OA with palmitic acid.

**Fig. 3:**
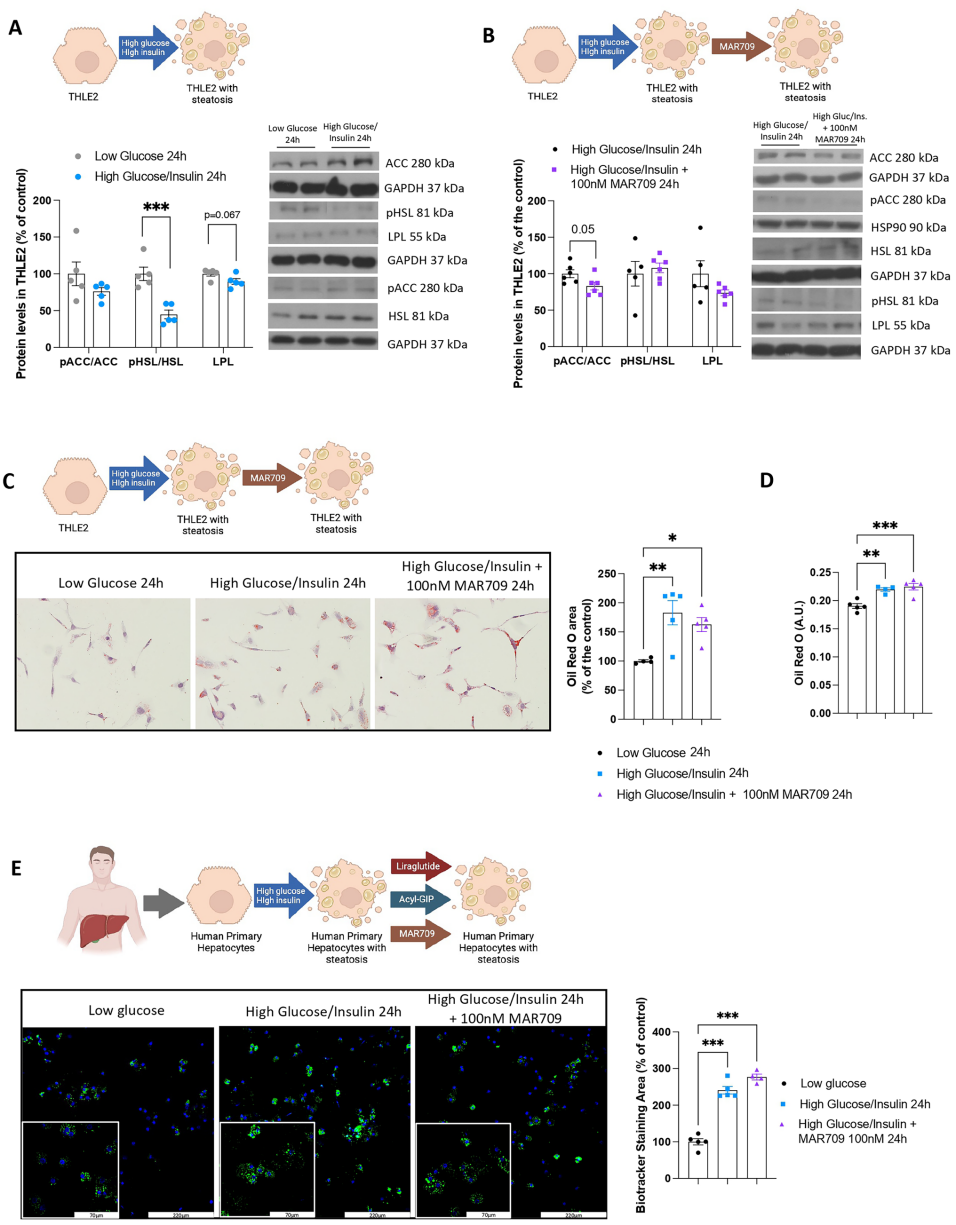
Effects of dual agonist MAR709 in THLE2 cells under high glucose and high insulin enviroment. **A** Protein levels of pACC, ACC, pHSL, HSL and LPL in THLE2 cells treated with KHH low glucose (6.25 mM, 24 h) and high glucose (25 mM, 24 h), high insulin (100 nM, 24 h) (n = 5). **B** Protein levels of pACC, ACC, pHSL, HSL and LPL in THLE2 cells treated KHH high glucose (25 mM, 24 h), high insulin (100 nM, 24 h) with and without dual agonist MAR709 (100 nM, 24 h) (n = 5–6). **C**, **D** Oil red O staining in THLE2 cells treated with KHH low glucose (6.25 mM, 24 h) and KHH high glucose (25 mM, 24 h), high insulin (100 nM) with and without dual agonist MAR709 (100 nM, 24 h) and Oil Red O quantification by spectrophotometry, respectively (n = 4–5). **E** Biotracker staining in human primary hepatocytes treated with low glucose (6.25 mM, 24 h) and KHH high glucose (25 mM, 24 h), high insulin (100 nM, 24 h) with and without dual agonist MAR709 (100 nM, 24 h). Data are presented as mean ± SEM; *p < 0.05, **p < 0.01, ***p < 0.001, using one-way ANOVA followed by a Bonferroni’s multiple comparison test

A steatosis mechanism highly pertinent to MASLD is de-novo lipogenesis, and MASLD is commonly accompanied by hyperglycemia and hyperinsulinemia. For instance, it has been demonstrated that GLP-1 decreases lipotoxicity in people with non-alcoholic steatohepatitis [4]. Therefore, we modeled this situation by performing an experiment using THLE2 cells under these conditions and measured enzymes involved in lipid metabolism. In a medium with high glucose/high insulin, the ratio of phosphorylated acetyl CoA carboxylase (ACC)/total ACC, remained unchanged (Fig. 3A). Moreover, the ratio of phosphorylated hormone sensitive lipase (pHSL)/total HSL was reduced, suggesting reduced lipid mobilization (Fig. 3A); and lipoprotein lipase (LPL), which stimulates lipid uptake, showed a tendency to be diminished but without significant differences (Fig. 3A). We subsequently incubated hepatocytes with high glucose/high insulin and treated them with MAR709. This treatment failed to modify the ratio of pACC/ACC, pHSL/HSL or protein levels of LPL (Fig. 3B), and consistently, the increased lipid content observed in cells with high glucose/high insulin was unaffected by the dual-agonist (Fig. 3C, D). Furthermore, we conducted the same experiment with high glucose/high insulin in primary human hepatocytes and treated them with MAR709, and we did not observe changes in lipid accumulation as marked by Biotracker (Fig. 3E).

**Fig. 4:**
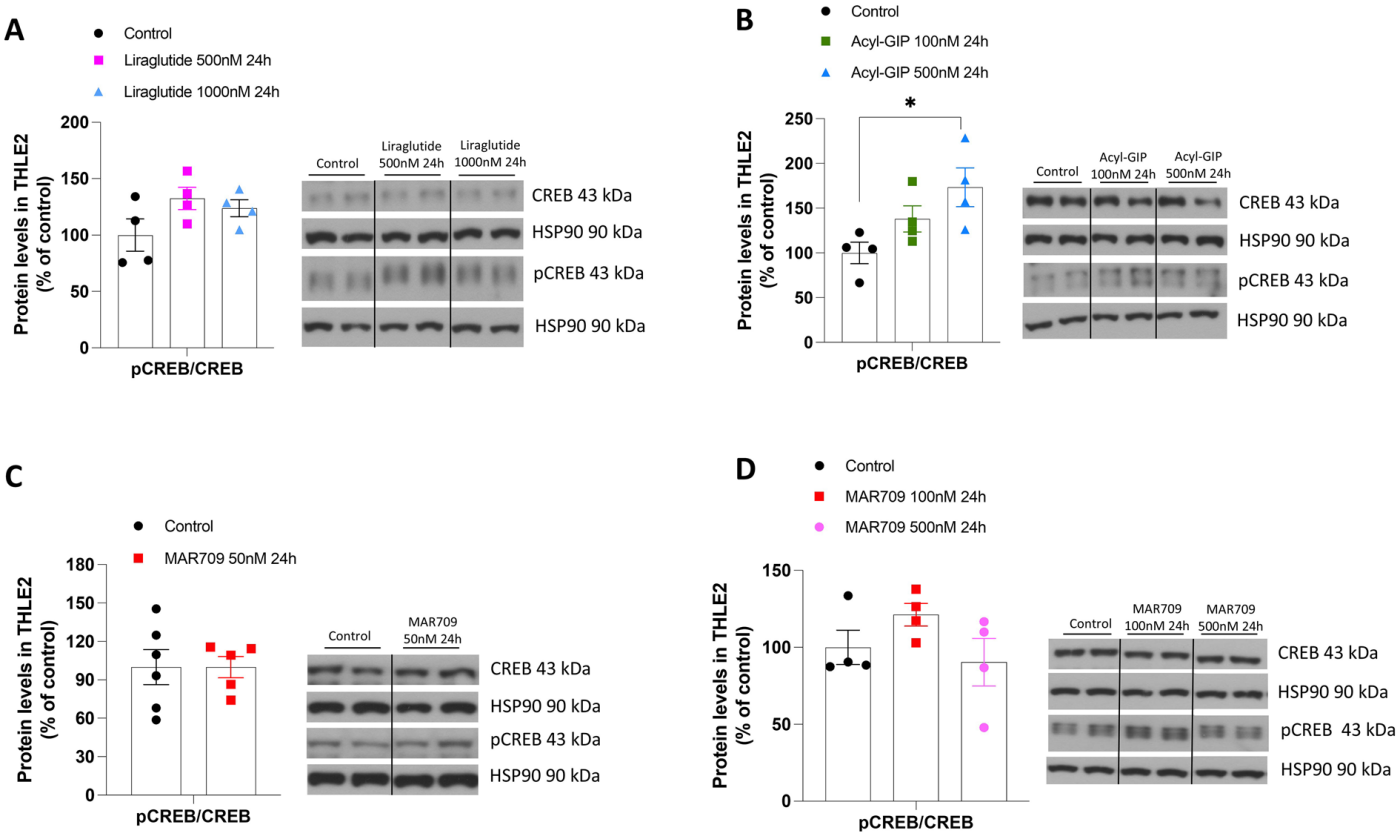
Effects of Liraglutide, Acyl GIP and GIP/GLP-1 dual agonist in human hepatocytes cells on protein levels. **A** Protein levels of CREB and pCREB in THLE2 cells treated with Liraglutide (500 and 1000 nM, 24 h) (n = 4). **B** Protein levels of CREB and pCREB in THLE2 cells treated with Acyl-GIP (100 and 500 nM, 24 h) (n = 4). **C**, **D** Protein levels of CREB and pCREB in THLE2 cells treated with MAR709 (50, 100 and 500 nM, 24 h) (n = 4). Protein levels were normalized using HSP90. Data are presented as mean ± SEM; *p < 0.05, using one-way ANOVA followed by a Bonferroni’s multiple comparison test

As it is well known that both GIP and GLP-1 require CREB to elicit different effects in beta-cells [20, 42], we also measured the ratio pCREB/CREB in hepatocytes treated with Liraglutide, Acyl-GIP or the GLP-1/GIP dual agonist. We found that pCREB/CREB remined unchanged after the treatment with the three compounds, except for the highest concentration of Acyl-GIP that induced the pCREB/CREB ratio (Fig. 4A–C). Overall, these data indicate that agonism of GLP-1R or GIPR, individually or in combination, does not affect fatty acids in hepatocytes in response to OA and the combination of OA with palmitic acid or in conditions of hyperglycemia and hyperinsulinemia.

### GLP-1R or GIPR agonism does not blunt TGFβ-induced HSCs activation

**Fig. 5:**
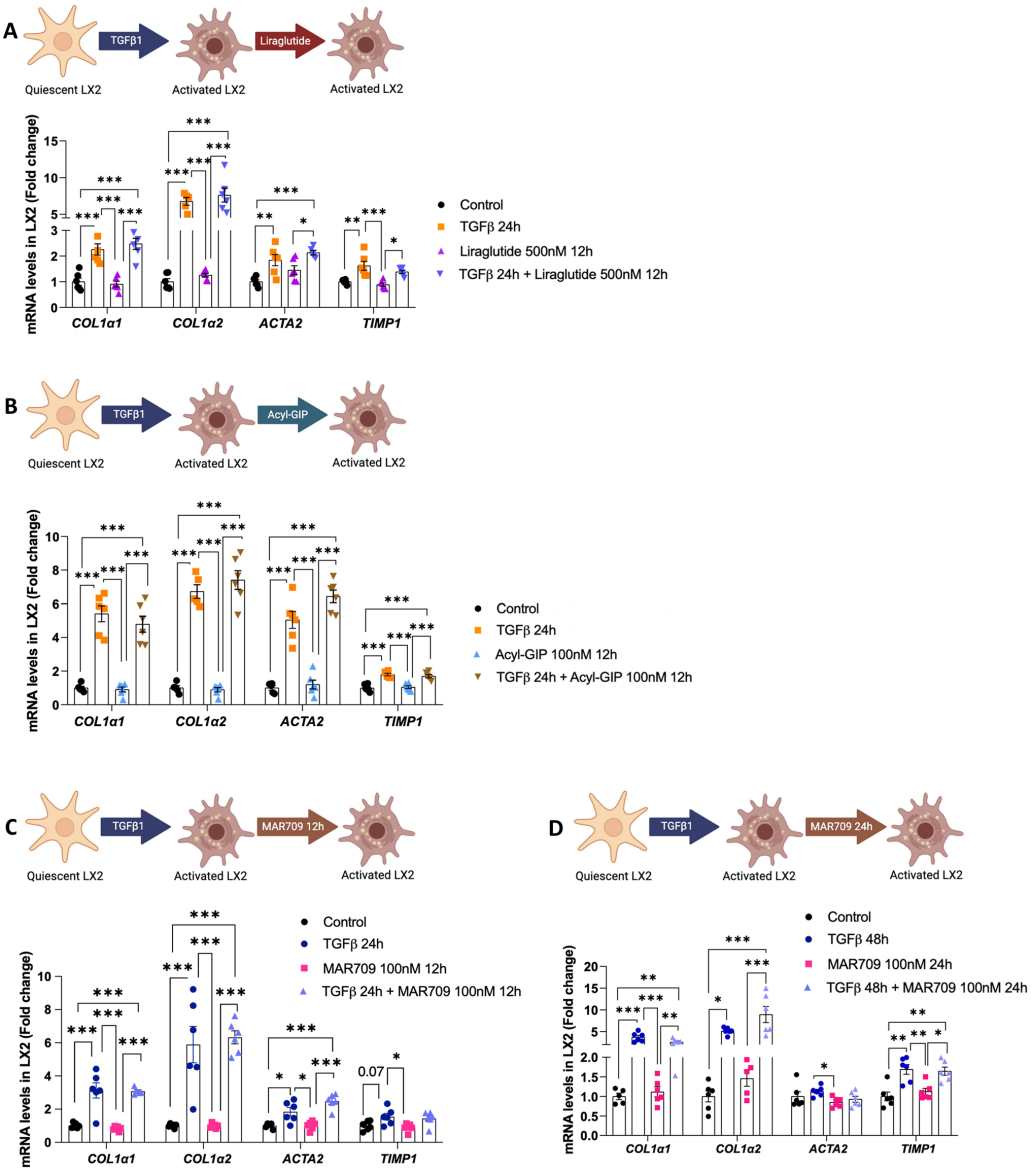
Effects of Liraglutide, Acyl-GIP and GIP/GLP-1 dual agonist in human hepatic stellate cells (LX2) on fibrosis onset. **A** mRNA expression of fibrotic markers in LX2 treated with TGFβ (8 ng/mL, 24 h) and with Liraglutide (500 nM, 12 h) (*n* = 5–6). **B** mRNA expression of fibrotic markers in LX2 treated with TGFβ (8 ng/ml, 24 h) and with Acyl-GIP (100 nM, 12 h) (*n* = 5–6). **C**, **D** mRNA expression of fibrotic markers in LX2 treated with TGFβ (8 ng/ml, 24 h) and with MAR709 (100 nM, 12 h) (*n* = 5–6) and with TGFβ (48 h) and with MAR709 (100 nM, 24 h) (*n* = 5–6), respectively. mRNA levels were normalized to the housekeeping gene HPRT. Data are presented as mean ± SEM; *p < 0.05, **p < 0.01, ***p < 0.001, using a one-way ANOVA followed by a Bonferroni’s multiple comparison test

Next, we studied whether these compounds could play a role in the activation of HSCs. For this, we treated LX2 -human hepatic stellate cell line- with transforming growth factor-b (TGFβ), a potent fibrogenic inducer, alone or in combination with Liraglutide, Acyl-GIP or the GLP-1/GIP dual agonist. While TGFβ increased the expression of the pro-fibrotic markers *COL1a1*, *COL1a2*, *ACTA2*, and *TIMP1*, none of the concentrations of each compound could alleviate the fibrotic action of TGFβ (Fig. 5A–D).

**Fig. 6:**
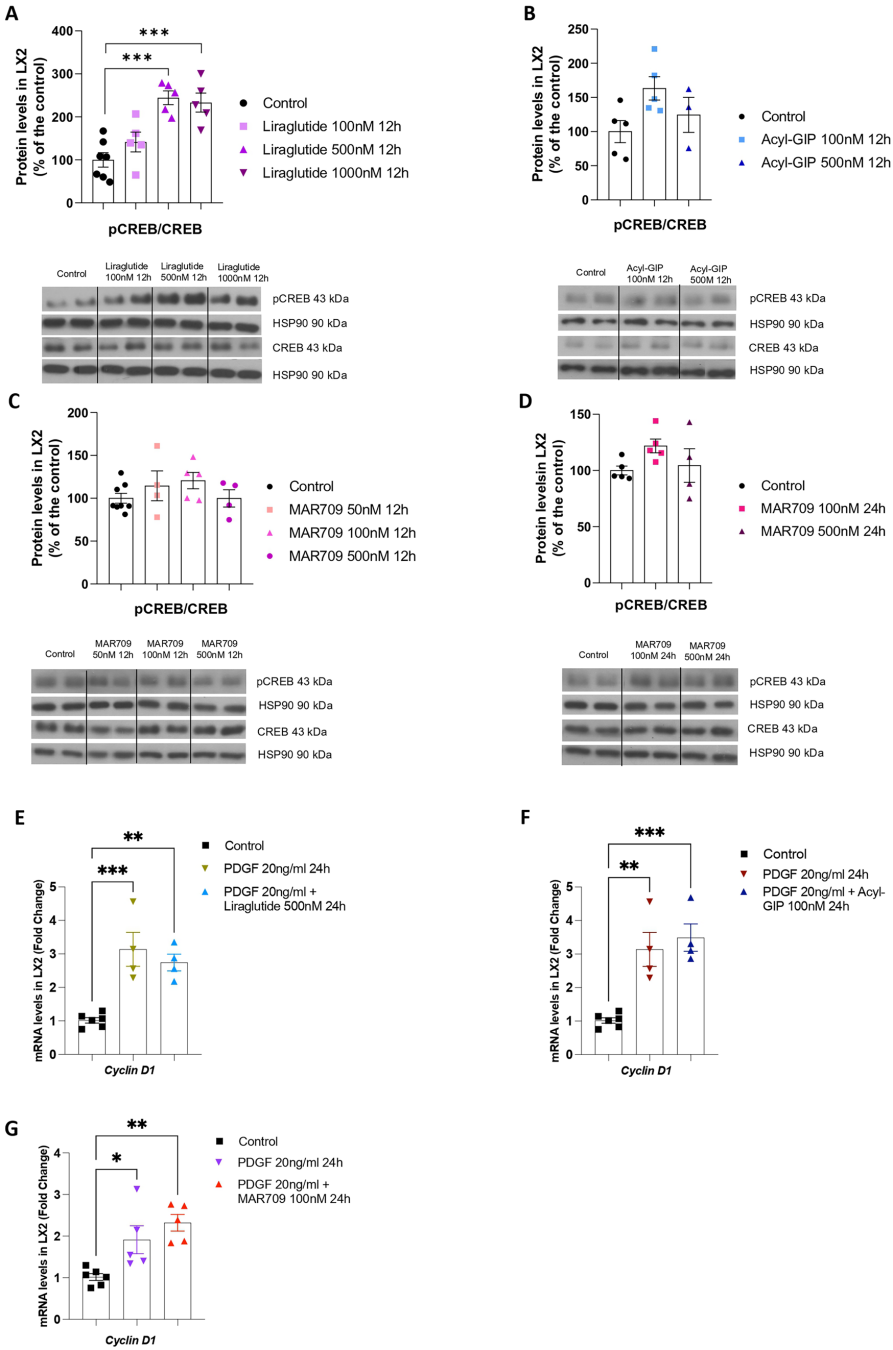
Effects of Liraglutide, Acyl-GIP and GIP/GLP-1 dual agonist in human hepatic stellate cells (LX2). **A** Protein levels of CREB and pCREB in LX2 cells treated with Liraglutide (100 nM, 500 nM and 1000 nM, 12 h) (*n* = 5–7). **B** CREB and pCREB protein levels in LX2 cells treated with Acyl-GIP (100 and 500 nM, 12 h) (*n* = 3–5). **C** Protein levels of CREB and pCREB in LX2 cells treated with MAR709 (50 nM, 100 nM and 500 nM, 12 h) (*n* = 4–7). **D** Protein levels of CREB and pCREB in LX2 cells treated with GIP/GLP-1 dual agonist (100 nM and 500 nM, 24 h) (*n* = 4–5). **E** mRNA expression of cyclin D1 in LX2 cells treated with PDGF (20 ng/ml) and Liraglutide (500 nM, 12 h) (*n* = 4–6). **F** mRNA expression of cyclin D1 in LX2 cells treated with PDGF (20 ng/ml) and Acyl-GIP (100 nM, 12 h) (*n* = 4–6). **G** mRNA expression of cyclin D1 in LX2 cells treated with PDGF (20 ng/ml) and MAR709 (100 nM, 24 h) (*n* = 5–6). mRNA levels were normalized to the housekeeping gene HPRT, and protein levels were normalized using HSP90. Data are presented as mean ± SEM; *p < 0.05, **p < 0.01, ***p < 0.001, using a one-way ANOVA followed by a Bonferroni’s multiple comparison test

We also assessed pCREB/CREB in HSCs treated with Liraglutide, Acyl-GIP or MAR709. While Liraglutide increased the pCREB/CREB ratio at high concentrations (500 and 1000 nM) (Fig. 6A), neither Liraglutide at 100 nM nor the other compounds at any concentration elicited any effect (Fig. 6A–D).

Platelet-derived growth factors (PDGFs) are cysteine knot–type growth factors that play a key role in mediating the activation and profibrogenic transdifferentiation of HSCs into myofibroblasts during hepatic fibrosis [21]. For instance, HSC-specific PDGF receptor α loss results in early reduction of fibrosis and HSC migration in a model of hepatotoxic liver injury, given the increase in HSC and myofibroblast cell death [19]. Therefore, we also studied whether the incretins could ameliorate the effect of PDGF. For this, we incubated LX2 cells with PDGF and each compound, and subsequently measured the expression of the cell cycle regulating gene cyclin D1. While PDGF induced the expected increase in cyclin D1 mRNA expression, neither Liraglutide, Acyl-GIP or MAR709 produced any effect (Fig. 6E–G).

**Fig. 7:**
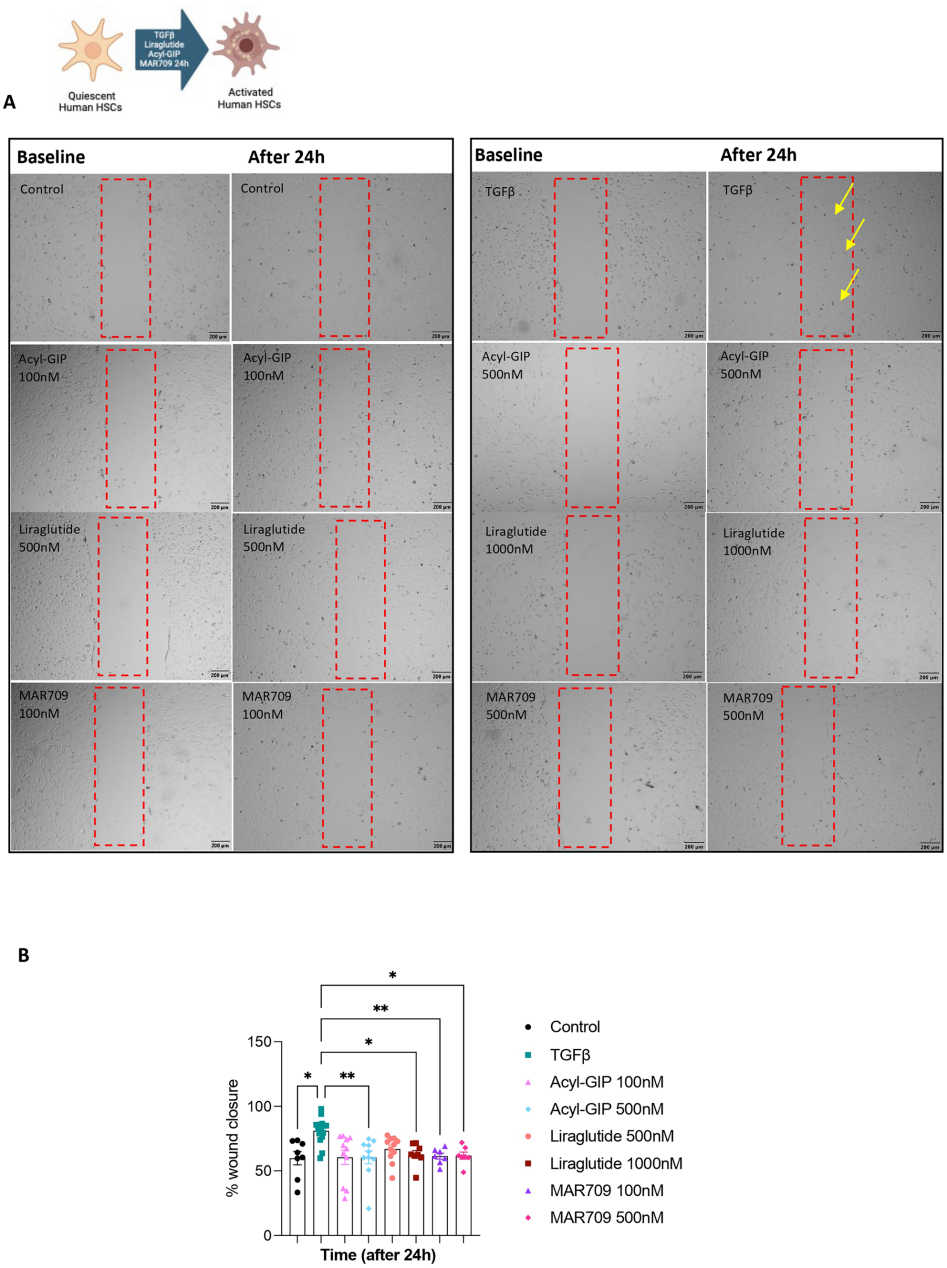
Effects of Liraglutide, Acyl-GIP and GIP/GLP-1 dual agonist on migration in human hepatic stellate cells (LX2). **A** Representative images of the scrape wound generated in the cell layer and then treated with Liraglutide (500 nM and 1000 nM, 24 h) (n = 10), Acyl-GIP (100 nM and 500 nM, 24 h) (*n* = 10) or MAR709 (100 nM and 500 nM, 24 h) (*n* = 8). TGFβ (8 ng/ml, 24 h) was used as a positive control. **B** Quantification of the % of wound closure after 24 h of treatment with each compound, Data are presented as mean ± SEM; *p < 0.05, **p < 0.01, ***p < 0.001, using a one-way ANOVA followed by a Bonferroni’s multiple comparison test

Moreover, to corroborate that Liraglutide, Acyl-GIP or MAR709 did not affect the migration of HSCs, we conducted a wound healing assay. While TGFβ treatment showed a notable impact on wound closure, the incubation with Liraglutide (500 nM and 1000 nM, 24 h), Acyl-GIP (100 nM and 500 nM, 24 h), and MAR709 (100 nM and 500 nM, 24 h) did not elicit any significant effect on the wound healing process (Fig. 7).

**Fig. 8:**
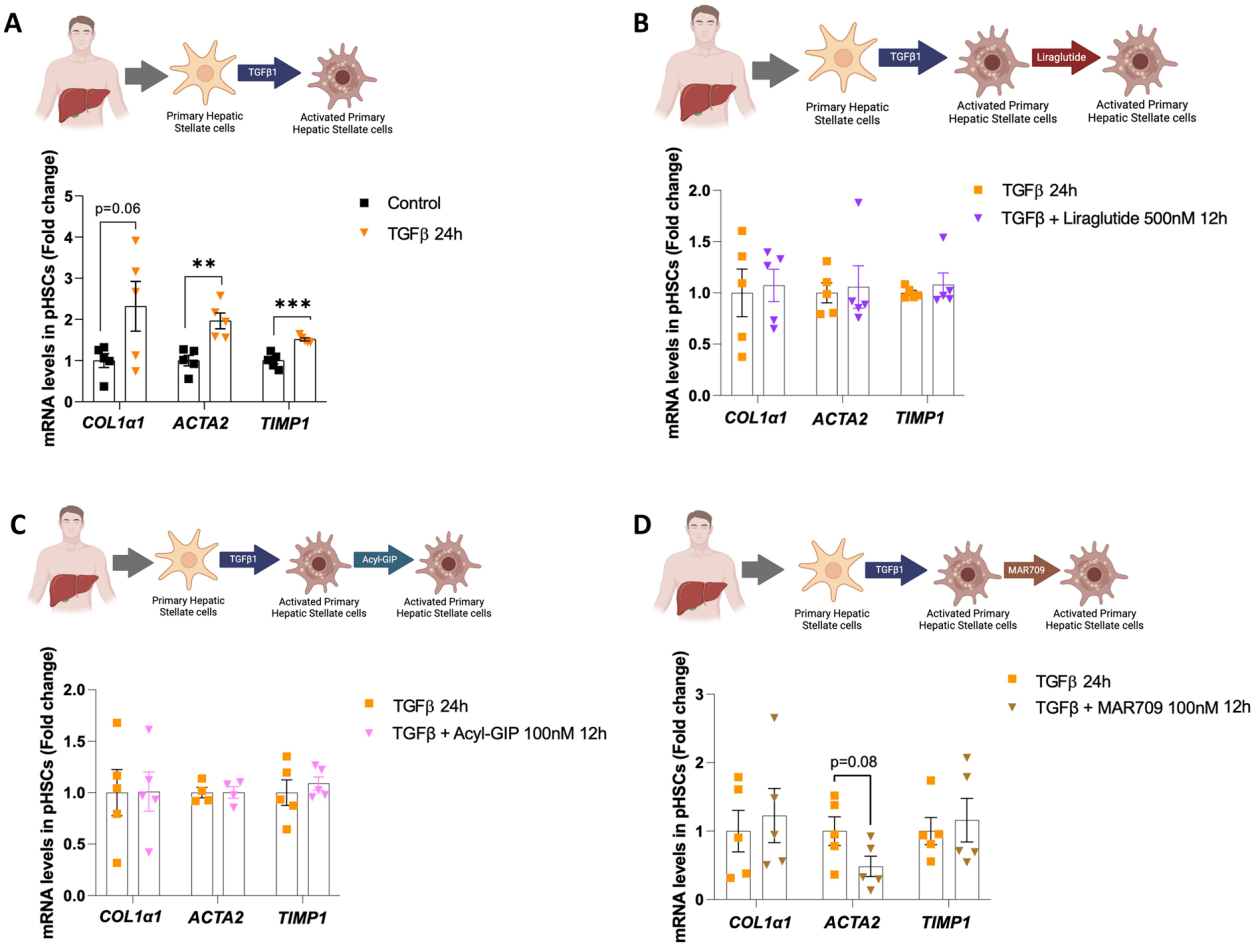
Effects of Liraglutide, Acyl-GIP and GIP/GLP-1 dual agonist in primary human hepatic stellate cells on fibrosis onset. **A** mRNA expression of fibrotic markers in human primary hepatic stellate cells (pHSCs) treated with TGFβ (8 ng/ml, 24 h) (*n* = 4–5). **B** mRNA expression of fibrotic markers in pHSCs treated with TGFβ (8 ng/ml, 24 h) and TGFβ with Liraglutide (500 nM, 12 h) (*n* = 5). **C** mRNA expression of fibrotic markers in pHSCs treated with TGFβ (8 ng/ml, 24 h) with or without Acyl-GIP (100 nM, 12 h) (*n* = 5). **D** mRNA expression of fibrotic markers in pHSCs treated with TGFβ (8 ng/ml, 24 h) and TGFβ with MAR709 (100 nM, 12 h) (*n* = 5). mRNA levels were normalized to the housekeeping gene HPRT. Data are presented as mean ± SEM; **p < 0.01, ***p < 0.001, using a t-test

To further investigate the efficacy of the compounds, we extended our study to primary human HSCs. We decided to evaluate both the direct effects of MAR709 and its potential to modulate TGFβ-induced activation in these cells. First, we incubated primary human HSCs with MAR709 alone for 24 h. Subsequent analysis revealed no statistically significant changes in the expression of typical activation markers (Supplementary Fig. 5A). Then, we stimulated the HSCs with TGFβ for 24 h to induce activation. As expected, this treatment resulted in a marked upregulation of fibrotic markers (Fig. 8A). Next, we treated primary human HSCs with TGFβ together with Liraglutide, Acyl-GIP or MAR709. However, despite a trend towards lower *ACTA2* expression in cells treated with the dual agonist, none of these compounds showed capacity to reduce TGFβ-induced levels of fibrotic markers (Fig. 8B–D).

Together, these results indicate that agonism of GLP-1R or GIPR, individually or in combination, does not regulate HSCs activation.

## Discussion

Among different clinical trials, both GLP-1RAs and GLP-1/GIP dual agonists are showing positive effects in reducing liver fat content and reversion of MASH [12, 38]. However, the ongoing discussion revolves around whether GLP-1RAs exert direct standalone effects on MASH or if their impact on pathophysiology arises from improvements in weight, insulin resistance, and glycemic control [31, 47]. In preclinical models, whereas several studies found low expression of *Glp1r* from mouse liver [43], others failed to detect *Glp-1r* mRNA transcripts in liver and isolated hepatocytes [22, 23]. The latter was further corroborated in diet-induced liver steatosis animal models [32]. In humans, the hepatic expression of *GlP-1R* has also been questioned, even though earlier studies have reported its presence in human hepatocytes, and suggested that *GLP1R* decreased hepatic steatosis by modulating the insulin signaling pathway [16] or activating lipid oxidation [43]. Indeed, these discrepancies might be explained by not using properly validated reagents for endogenous GLP-1R/GIPR detection [5]. Our search in different databases indicated that *GLP1R* could not be found in human liver cells, at least at meaningful expression levels. The presence of GIPR in the liver is also controversial. While *Gipr* was not found in the liver of rodents [45], GIP enhances lipid deposition in liver and inhibition of GIP signal prevents this process [13, 30, 33]. Similarly to *GLP1R*, our search in different databases failed to detect a representative *GIPR* expression in liver cells.

Despite these receptors could not be detected in liver cells, numerous studies in mice and some clinical trials have reported that GLP-1RAs [2, 6] and the co-agonism of GLP-1R and GIPR [12] reduce liver fat content. However, there is the possibility that these agonists might work through an extremely small population of receptors that could be present in these cells at levels below the quantitative limits of the transcript measurements. In addition, GLP-1R and GIPR belong to family B of the seven transmembrane G-protein coupled receptors that include receptors for glucagon-like peptide-2 (GLP-2), GIP, vasoactive intestinal polypeptide (VIP), pituitary adenylate cyclase-activating polypeptide (PACAP), GHRH, secretin, calcitonin, corticotropin releasing hormone (CRH) or PTH. The N-terminal extracellular domain of family B receptors is important for selective ligand interaction however, the extracellular loops and the extracellular end of the transmembrane segments can provide additional determinants of ligand selectivity [40]. These receptors are known to play different functions in liver metabolism. For example, VIP receptor 1 is found in the liver [7], and a high-fat diet increases its hepatic expression [36]. PACAP receptors have been detected in the liver, and PACAP attenuates hepatic lipid accumulation during overnutrition [27]. Additionally, PACAP-null mice developed microvascular fat accumulation in the liver, skeletal muscle, and heart, and displayed significantly higher serum triglycerides and cholesterol levels than littermates [15]. Secretin (Sct) is an important homeostatic regulator of pancreatic and liver secretory function, as it binds to its receptors on large cholangiocytes. Sct secretion increases biliary mass and liver fibrosis [14]. Calcitonin receptor agonists have demonstrated robust body weight loss, improved glucose tolerance and a decreased deposition of fat in liver tissue beyond what is observed after a body weight loss. relevant metabolic effects in the context of MASLD [29].

Therefore, we assessed whether the agonists of GLP-1R and GIPR could act directly in the liver. More specifically, we tested two liver cell types that are strongly linked to the progression of MASH. For instance, hepatocytes are central to the understanding of the disease since these cells accumulate fat in the early stages of the disease and suffer different processes such as inflammation, oxidative stress, and cellular damage. Moreover, activation of HSCs upon liver injury induces their transformation into myofibroblast-like cells, which are the main producers of extracellular matrix proteins and leads to the formation of fibrous scar tissue within the liver.

Our results indicate that individual or dual agonism of GLP-1R and GIPR could not prevent the high fatty acid content induced by OA- or the combination of OA together with palmitic acid in hepatocytes. The TGFβ-induced activation of HSCs remained also unaffected after the treatment with the three compounds. Importantly, the selected concentrations for each compound have been selected according to previous reports [8, 48, 51] and we tested that they were efficient in terms of insulin secretion from beta cells. Although we cannot discard the role of other cell types residing in the liver different than hepatocytes and HSCs, the low expression of *GLP1R* and *GIPR* in hepatic cell types (according to datasets), makes it unlikely that those cell types could be responsible for the entire beneficial effects of the GLP-1R/GIPR dual-agonism in people with MASH.

## Conclusions

Our findings demonstrate that individual or dual agonism of GLP-1R/GIPR has no direct actions in hepatocytes or HSCs, which might suggest that the effects of dual agonism on liver function are mediated by the reduction of weight loss and enhancement in insulin sensitivity.

## Supplementary Information

Below is the link to the electronic supplementary material.

Supplementary file1 (PDF 1380 KB)

Supplementary file2 (PDF 286 KB)

## Data Availability

The data supporting the findings of this study are found within the article and the supplementary material. All relevant raw data will be made available from the corresponding author upon reasonable request.
