# Supplementary material for: GLP-1 and GIP agonism has no direct actions in human hepatocytes or hepatic stellate cells
Source: Cell Mol Life Sci. 2024 Nov 28;81(1):468. doi: 10.1007/s00018-024-05507-6 (PMC11604888; doi:10.1007/s00018-024-05507-6)
Supplement: Supplementary file 1 — Supplementary file1 (PDF 1380 KB) [file 18_2024_5507_MOESM1_ESM.pdf]

Material and methods

1.1. Public bulk RNA-sea and scRNA-seq datasets analysis

Raw counts of bulk RNA-seq dataset were obtained from the public repository GSE135251. Differential expression analysis was performed in DESeq2 v1.34.0 (R v4.1.3). Gene expression results from Human Liver Cell Atlas were obtained through the Atlas web tool (https://www.livercellatlas.org/umap-humanAll.php). Human Single-Nucleus RNA sequencing data from healthy, cirrhotic and HCC livers were obtained from GEO accessions: GSE185477, GSE174748 and GSE212046. The Seurat package (v4.3.0) in R was used for subsequent analysis of the 10X CellRanger count outputs obtained from Gene Expression Omnibus. For quality control, nuclei with mitochondrial content > 30% were removed. Principal component analysis was performed using the top 3,000 most variable genes and UMAP analysis was performed with the top 20 PCAs. Clustering was performed using a resolution of 0.5. For cell annotation we use the Azimuth Human Liver Reference (https://azimuth.hubmapconsortium.org/references/#Human%20-%20Liver).

1.2.Cell line cultures

THLE2 human hepatic cell line (CRL-2706, ATCC) was cultured in bronchial epithelial cell basal medium (BEBM) supplemented with a growth factors BulleKit (Lonza/Clonetics Corporation), 70 ng/mL phosphoethanolamine, 5 ng/mL epidermal growth factor, 10% (v/v) FBS and 1% (v/v) Glutamine-Penicillin-Streptomycin solution (MERCK). THLE2 cells were grown on culture plates pre-coated with a mixture of 0.01 mg/ml fibronectin (#33010018, Sigma Aldrich), 0.01 mg/ml bovine serum albumin (#A4503, Sigma Aldrich) and 0.03 mg/ml collagen type I (#sc-136157, Santa Cruz).

The LX2 human hepatic stellate cell line (SCC064, MERCK) was grown in Dulbecco's Modified Eagle's Medium (DMEM) high glucose (D5796, MERCK) supplemented with 2% (v/v) FBS and 1% (v/v) Penicillin-Streptomycin solution. Cell lines were maintained at 37ºC in a humidified atmosphere containing 5% CO_2.

The INS1 rat insulinoma cell line (SCC207, MERCK) was grown in RPMI 1540 (Sigma Aldrich) supplemented with 2mM L-glutamine (TMS-002-C), 1mM sodium pyruvate (TMS-005-B), 10mM HEPES (TSM-003-C), 0.05mM β-Mercaptoethanol (TSM-007-C), 10% (v/v) FBS, 1% (v/v) Penicillin-Streptomycin solution. Cell lines were maintained at 37ºC in a humidified atmosphere containing 5% CO_2.

The MCF-10A cell line (CRL-10317, ATCC) was cultured in DMEM/F12 (Sigma-Aldrich) supplemented with 5% horse serum (Stem Cell Technologies), 0.5% penicillin/streptomycin (Sigma-Aldrich), 10µg/ml insulin (Thermo Fisher Scientific), 0.5µg/ml hydrocortisone (Stem Cell Technologies), 20ng/ml hEGF (Sigma-Aldrich) and 100ng/ml cholera toxin (Sigma-Aldrich). Cell lines were maintained at 37ºC in a humidified atmosphere containing 5% CO_2.

1.3.Human Primary Hepatocytes and Human Primary Hepatic Stellate Cells

Human primary hepatocytes (NHM2354B-HE-N, BeCytes Biotechnologies) were thawed using Hepatocyte Thawing Medium (MHT-23-14, BeCytes Biotechnologies) according to the manufacturer indications. After centrifugation, cells were resuspended and seeded with Hepatocyte Plating Medium (HPM-250, BeCytes Biotechnologies). After six hours, the medium was changed to Hepatocyte Maintenance Medium (MHM-250, BeCytes Biotechnologies) supplemented according to the different treatments. Primary cells were maintained at 37ºC in a humidified atmosphere containing 5% CO_2.

Human primary hepatic stellate cells (CyHum19007-SC-P2-Z, BeCytes Biotechnologies) were thawed using the Thawing medium (MHT-NPC50, BeCytes Biotechnologies) according to the manufacturer’s instructions. Afterwards, cells were seeded and maintaining using Growth medium for Stellate cells (HMSG-50, BeCytes Biotechnologies). Primary cells were maintained at 37ºC in a humidified atmosphere containing 5% CO_2.

1.4.Oleic and palmitate acid experiments

In the study with oleic acid, 0.5 x 10^5 THLE2 cells and human primary hepatocytes were seeded in a twenty-four-well plate. Control and treated cells were exposed to FBS-free medium supplemented with 1 mM of oleic acid (MERCK) bound to fatty acid-free BSA (Capricorn) at a 2:1 molar ratio or 0.25mM Oleic Acid + 0.125mM Palmitate for 24 hours to induce lipid accumulation. Controls were supplemented with fatty acid-free BSA alone. The treatment with Liraglutide for GLP-1R (500nM and 1000nM), Acyl-GIP for GIPR (100nM and 500nM) or the dual agonist (50nM, 100nM and 500nM) was added after 24 hours of Oleic Acid 1mM or Oleic Acid 0.25mM + Palmitate 0.125mM. Cells were stained with Oil Red O to detect lipids.

0.3 x 10^5 human primary hepatocytes were seeded in 24-well plate using Hepatocyte Plating Medium and cultivated with Hepatocyte Maintenance Medium with or without 0.25mM Oleic Acid + 0.125mM Palmitate for 12 hours to induce lipid accumulation. The treatment with Liraglutide (500nM), Acyl-GIP (100nM) or the dual agonist MAR709 (100nM) were added for 24h to detect lipid accumulation by Biotracker Staining (BioTracker 488 Green Lipid Dye, Cat. #SCT144, Merck).

1.5.TGFβ treatment

0.5x10^5 LX2 cells were seeded in a 24-well plate and then incubated with medium supplemented with recombinant human TGFβ (8 ng/ml) (100-21, PreproTech) for 24 or 48 hours, to induce cell activation. Then, these cells were treated with Liraglutide (500nM 12h), acyl GIP (100nM 12h) or dual agonist (100nM 12 and 24h). Once treatments were completed, LX2 cells were collected for mRNA extraction.

0.8 x 10^4 human primary hepatic stellate cells were seeded in 24-well plate using Growth medium for Stellate cells with or without TGFβ (8 ng/ml) (100-21, PreproTech) for 24 to induce cell activation. The treatment with Liraglutide for GLP-1R (500nM), Acyl-GIP for GIPR (100nM) or the dual agonist MAR709 (100nM) were added for 12h. Afterwards, the cells were collected for mRNA expression studies.

1.6.Wound healing assay

LX2 cells were seeded at a density of 2.5x10^5 in a 6-well plate. Following a 48-hour incubation period, a linear scratch was created across the cell monolayer in each well using a sterile 10 µL pipette tip. The plate was then gently washed with phosphate-buffered saline (PBS) to remove detached cells and debris. After washing, fresh medium (with or without Acyl-GIP 100nM and 500nM, Liraglutide 500nM and 1000nM, and MAR709 100nM and 500nM) was added to each well. We used TGFβ (8ng/ml) as positive control. Images of the wound area were captured at the initial time point and after 24 hours using an inverted microscope (Olympus, IX73) to document changes in wound width and establish a baseline for wound closure. The wound areas were quantified using ImageJ 1.52p software to assess the extent of cell migration.

1.7.Glucose stimulated insulin secretion (GSIS)

INS-1 cells were seeded at a density of $0.5x10^6$ in a 24-well plate. After 2 days, the medium was changed, and the assay was performed on day 3. The glucose stimulated insulin secretion was performed in HBSS (Hepes balanced salt solution): 114 mmol/L NaCl, 4.7 mmol/L KCl, 1.2 mmol/L $KH2PO_4$, 1.16 mmol/L $MgSO_4$, 20 mmol/L HEPES, 2.5 mmol/L $CaCl_2$, 25.5 mmol/L $NaHCO_3$, and 0.2% bovine serum albumin, pH 7.2. Cells were cultured with HBSS + 2.5 mM glucose (normal conditions) or 15 mM glucose (hyperglycemia conditions) for 1 hour. After 1 hour, the secretagogues diluted in HBSS were added for 2 hours. After 2 hours, cells were fixed with PFA 4% for 15 minutes. For insulin quantification, INS-1 cells were seeded at a density of $1x10^6$ cells following the protocol described below, and the supernatant was subsequently collected for analysis using an ELISA kit.

1.8. Insulin levels quantification

For insulin levels quantification in supernatant of INS1 cells stimulated with HBSS and 2.5mM glucose (normal conditions) or 15 mM glucose (hyperglycemia conditions) and Acyl-GIP (100nM and 500nM), Liraglutide (500nM and 1000nM) or MAR709 (100nM and 500nM) we used ELISA kit Rat/Mouse according to the manufacturer's protocol (Millipore, EZRMI-13K). Volumes of 10 μL of samples (diluted 1:20) or standard were loaded to appropriate wells. Insulin (ng/mL) concentration was calculated by extrapolation of the data in a standard curve constructed with known concentrations of insulin.

1.9.Hyperglycemia-Hyperinsulinemia conditioned medium

THLE2 cells were seeded at a density of $0.3x10^6$ in a 6-well plate for molecular analysis and $0.5x10^5$ in a 24-well plate for Oil Red O staining. Cells were incubated in a medium depleted of nutrients Krebs-Henseleit-HEPES (KHH; 120 mmol l–1 NaCl, 4.7 mmol l–1 KCl, 2.5 mmol l–1 CaCl2, 1.2 mmol l–1 MgSO4, 1.2 mmol l–1 KH2PO4, 25 mmol l–1 NaHCO3, 25 mmol l–1 HEPES pH 7.4). To study lipogenesis in vitro, cells were cultured in KHH, with low (6,25 mM) or high glucose concentration (25 mM) (D-glucose, #G8270, Sigma-Aldrich) and high insulin (100 nM) (Actrapid, Novo Nordisk,), and with or without MAR709 (100nM) for 24h.

Primary human hepatocytes (NHM2354B-HE-N, BeCytes Biotechnologies) were seeded at a density of $0.4x10^5$ in a 24-well plate for Biotracker staining (BioTracker 488 Green Lipid Dye, Cat. #SCT144, Merck) using Hepatocyte Plating Medium (HPM-250, BeCytes Biotechnologies). After 6 hours, cells were changed to Hepatocyte Maintenance Medium (MHM-250, BeCytes Biotechnologies), in which we added up to 25mM glucose (D-glucose, #G8270, Sigma-Aldrich) and 2µM of insulin (Actrapid, Novo Nordisk,) and treated with or without Liraglutide (500nM), Acyl-GIP (100nM) and MAR709 (100nM) for 24h.

1.10. Immunofluorescence

After fixation with PFA 4% for 15 minutes, cells were washed with PBS and blocked with blocking buffer (5% donkey serum, 0.3% Triton X-100 and PBS 1x) for 1 hour at room temperature. Subsequently, cells were incubated with the respective primary antibodies (Anti-Insulin 05-1066, Merck; Anti-GLP-1R Mab 7F38, DSHB) diluted in the dilution buffer (1% BSA, 0.3% Triton X-100 and PBS 1x) in a humid chamber overnight at 4 ºC.

After this, cells were incubated with a secondary antibody 1:1000 (Alexa fluor 48, 115-545-003, Sigma Aldrich) in the dilution buffer. Finally, cells were mounted with a fluorescence-compatible medium (Fluoro-Gel with Tris buffer, Electron Microscopy Science, Cat #17985-10) with DAPI 1:1000 (D9542, Sigma-Aldrich) and subjected to microscopic examination. In this immunostaining technique, up to 6 representative microphotographs of each sample were taken with a Thunder Imager tissue microscope (Leica Microsystems). Leica Las X 3.7.4 software was used for the acquisition and analysis of immunohistochemistry/immunofluorescence staining. Image J 1.52p software was used for the quantification of the staining area.

1.11. Biotracker staining

After fixation with PFA 4% for 15 minutes, cells were washed with PBS and staining with Biotracker (BioTracker 488 Green Lipid Dye, Cat. #SCT144, Merck) in a humid chamber during 15 minutes at RT. After, primary human hepatocytes were mounted with a fluorescence-compatible medium (Fluoro-Gel with Tris buffer, Electron Microscopy Science, Cat #17985-10) with DAPI 1:1000 (D9542, Sigma-Aldrich) and subjected to microscopic examination. Five representative microphotographs of each sample were taken with a Thunder Imager tissue microscope (Leica Microsystems). Leica Las X 3.7.4 software was used to acquire and analyze immunohistochemistry/immunofluorescence staining. Image J 1.52p software was used for the quantification of the staining area.

1.12. PDGF treatment in LX2 cells

The LX2 human hepatic stellate cells were seeded and after 24h were submitted to starving using FBS-free RPMI medium during other 24h. Human recombinant PDGF-BB

(100-14B, PeproTech) was administered in LX2 cells at a concentration of 20 ng/ml [1] together the treatment with GLP-1R (500nM), GIPR (100nM) or dual agonism (100nM) [1]. Sterile water was used as vehicle.

1.13. Real-time PCR

RNA was extracted from cells samples using Trizol reagent (Invitrogen) according to the manufacturer's instructions. 100 ng of total RNA were used for each RT reaction, and cDNA synthesis was performed using the SuperScript First-Strand Synthesis System (Invitrogen) and random primers. Negative control reactions, containing all reagents except the sample were used to ensure specificity of the PCR amplification. For Real-time PCR, we used a fluorescent temperature cycler (Applied Biosystems) following the manufacturer’s instructions and TaqMan (Applied Biosystems, Life Technologies) for fibrotic markers. The TaqMan cycling conditions included an initial denaturation at 50 ºC for 10 min followed by 45 cycles at 95 ºC for 15 s and 60 ºC for 1 min. Specific Taqman probes were used to measure COL1α1 (Hs00164004_m1), COL1α2 (Hs01028956_m1), ACTA2 (Hs00426835_g1) and TIMP1 (Hs01092512_g1). All reactions were performed in duplicate using the QuantStudio 5 Real-Time PCR (qPCR). Expression levels were normalized to HPRT1 (Hs02800695_m1) for each sample and the fold change value was determined from the equation $2^{-\Delta\Delta Ct}$.

1.14. Western blot analysis

Total protein lysates from LX2 and THLE2 cells (6-9 µg, respectively) were subjected to SDS-PAGE, electrotransferred onto polyvinylidene difluoride membranes (BioRad) and

probed with the antibodies: phosphoCREB 1:1000 Rabbit (9198S, Cell Signaling), CREB 1:1000 Rabbit (4820S, Cell Signaling), ACC 1:1000 Rabbit (ab45174, Abcam), pACC 1:1000 Rabbit (3661S, Cell Signaling), phophoHSL 1:1000 Rabbit (45804S, Cell Signaling), HSL 1:1000 Rabbit (ab45422, Abcam), LPL 1:1000 Rabbit (SC32885, Santa Cruz), GAPDH 1:1000 Mouse (ab9485, Abcam) and HSP90 1:5000 Mouse (SC7947, Santa Cruz). For protein detection, horseradish peroxidase-conjugated secondary antibodies and chemiluminescence (Amersham Biosciences) were used. Membranes were exposed to radiograph film (Super RX Fuji Medical XRay Film, Fujifilm) and developed with developer and fixing liquids (AGFA) under appropriate dark room conditions. Protein expression was quantified by densitometric analysis with Image J software. Protein levels were normalized to GAPDH or HSP90 for each sample and expressed relative to the control group.

1.15. Statistical Analysis

Data are expressed as mean ± standard error mean (SEM). Statistical significance was determined by two-tailed Student's t-test or Mann-Whitney test when two groups were compared or One-way ANOVA with Bonferroni post-test. The significance level was set at $p<0.05$. GraphPad Prism Software 9.01 was used to compute the statistical analyses and graphics.

REFERENCES

1. Adachi T, Togashi H, Suzuki A, et al. NAD(P)H oxidase plays a crucial role in PDGF-induced proliferation of hepatic stellate cells. Hepatology. 2005;41(6):1272-81. Epub 2005/05/26.

Supplementary Figure 1

A

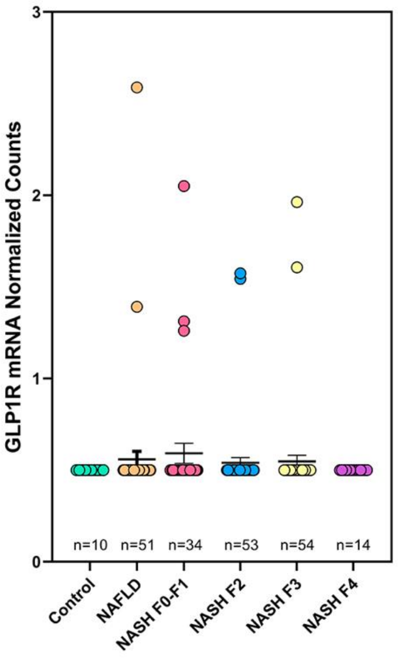

B

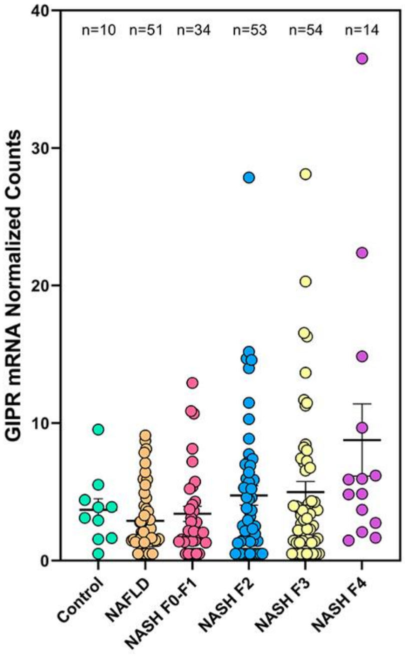

C

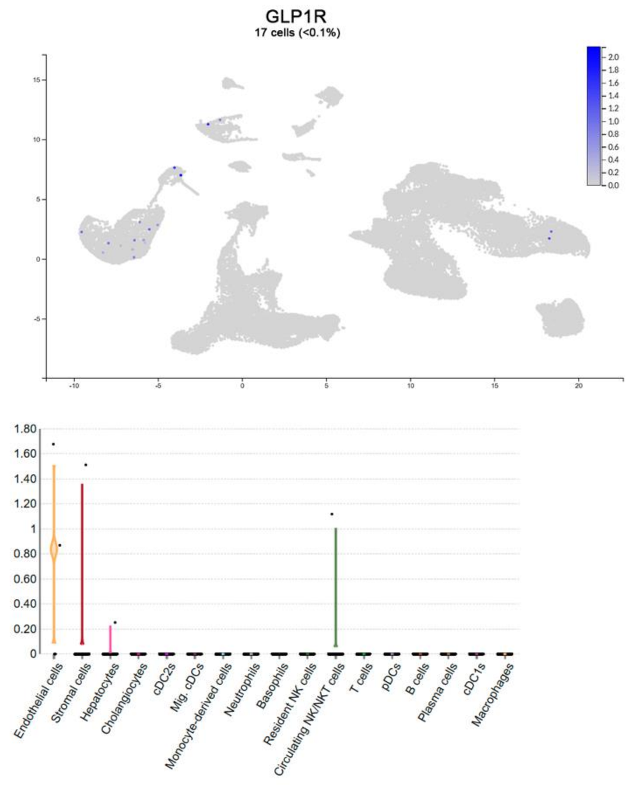

D

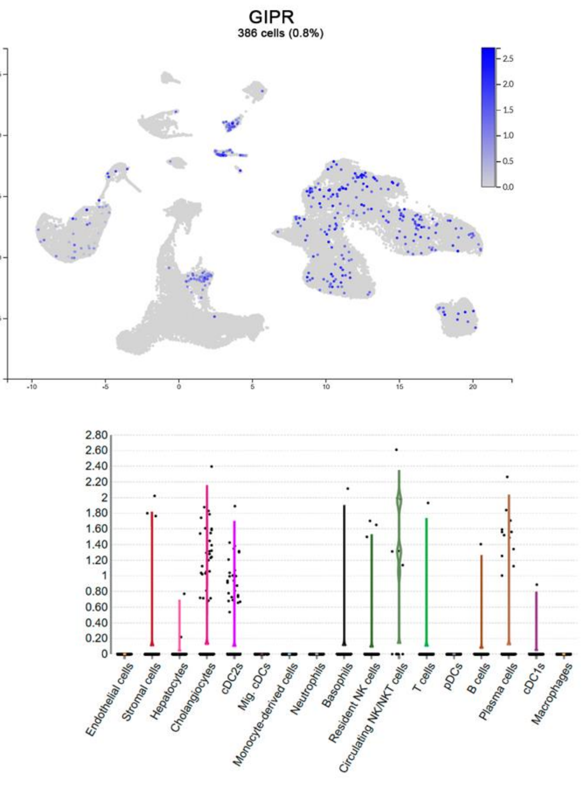

E

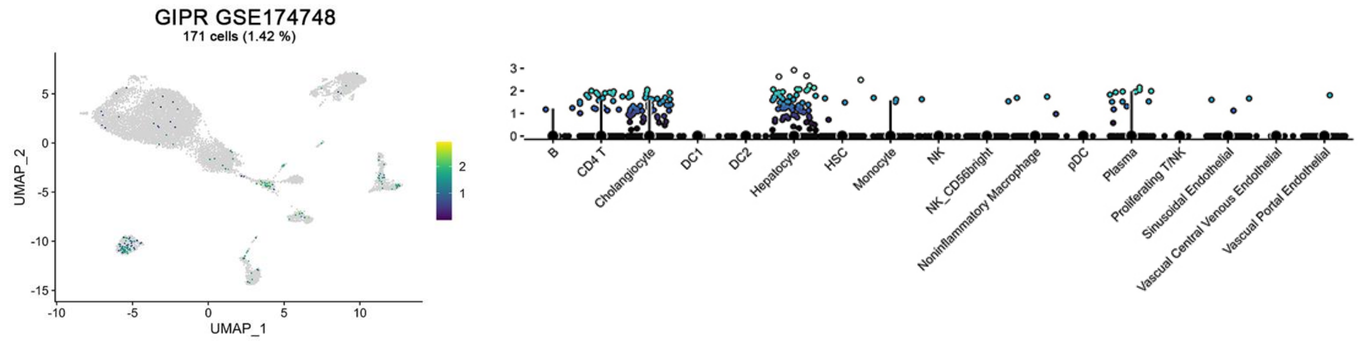

F

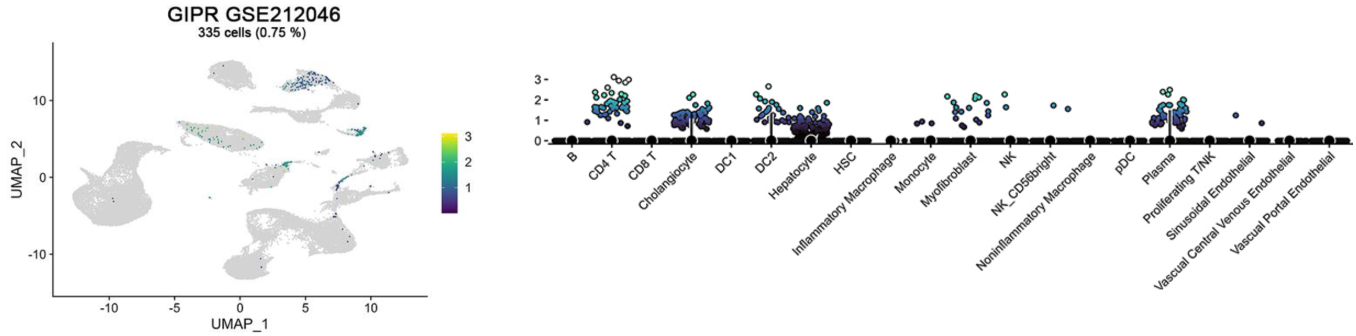

G

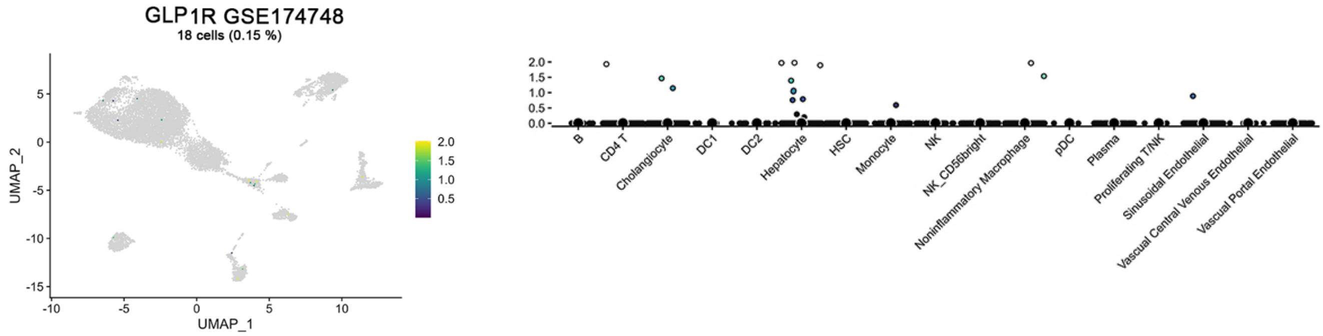

Supplementary Figure 1. Expression of *GLP1R* and *GIPR* in the liver of people with MASLD. (A-B) Normalised DESeq2 counts for the *GLP1R* (A) and *GIPR* (B) genes across the GSE135251 dataset composed of liver samples from control patients and patients with 5 grades of MASLD progression. (C-D) Feature Plots in the UMAP representation of the human dataset from the Liver Cell Atlas web resource together with Violin Plots showing *GLP1R* (C) and *GIPR* (D) expression in individual cells of the different cell types. Order dots were set in the Feature Plots to highlight the few positive cells. (E-G) Feature Plots in the UMAP representation of the human liver datasets GSE185477, GSE174748 and GSE212046 showing *GIPR* (E-F) and *GLP1R* (G) expression with their corresponding Geyser Plots showing the expression of the genes in the different cell types.

Supplementary Figure 2

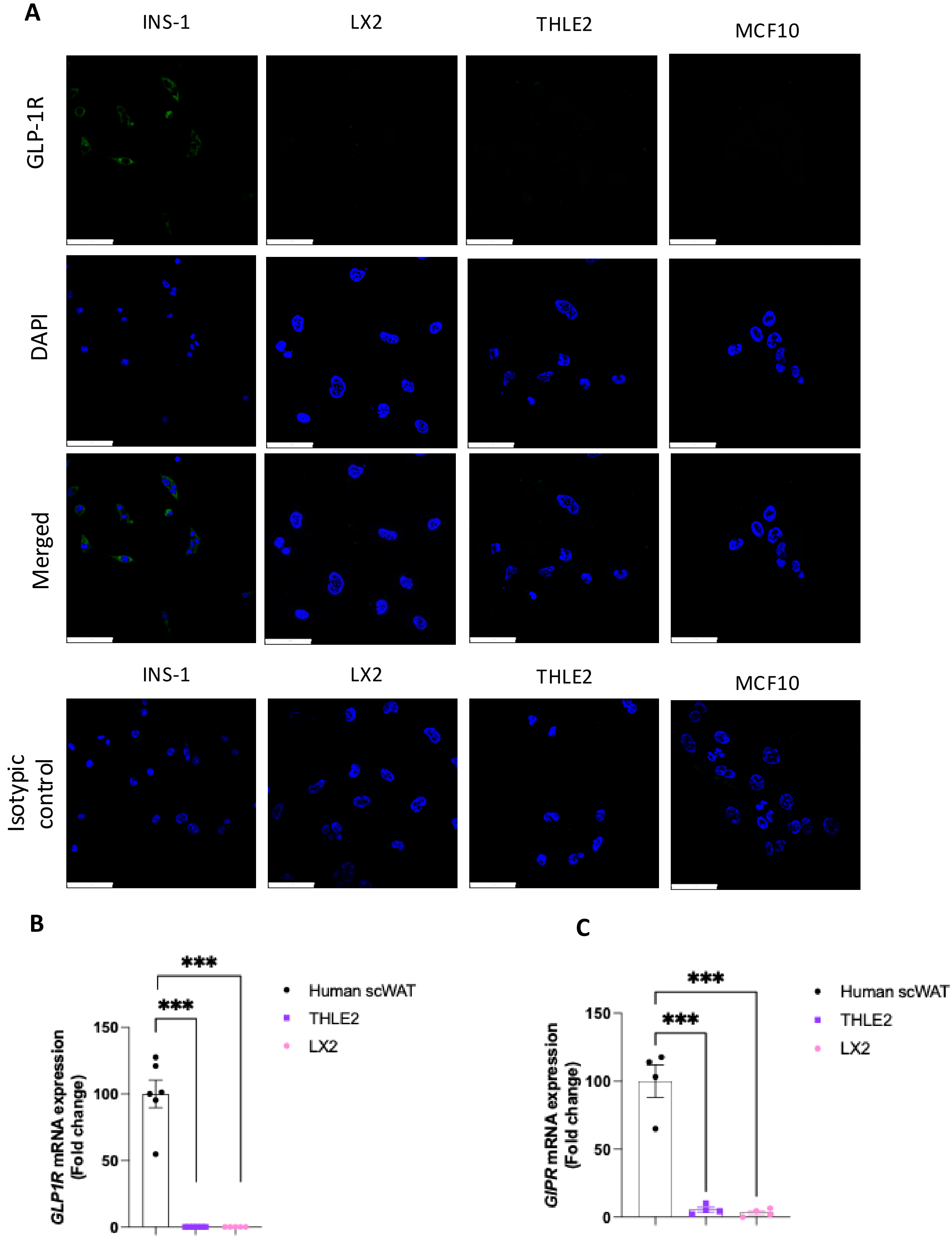

Supplementary Figure 2. Levels of *GLP1R* and *GIPR* in THLE2 and LX2 cells. (A) GLP-1R immunofluorescence representative images in INS-1, THLE2, LX2 and MCF10 cells. (B) *GLP1R* mRNA expression in human subcutaneous adipose tissue (scWAT), THLE2 and LX2 cells (*n*=6). (C) *GIPR* mRNA expression in human subcutaneous adipose tissue (scWAT), THLE2 and LX2 cells (*n*=4). Data are presented as mean ± SEM; *p < 0.05, **p < 0.01***, p < 0.001, using a one-way ANOVA followed by a Bonferroni's multiple comparison test.

Supplementary Figure 3

A

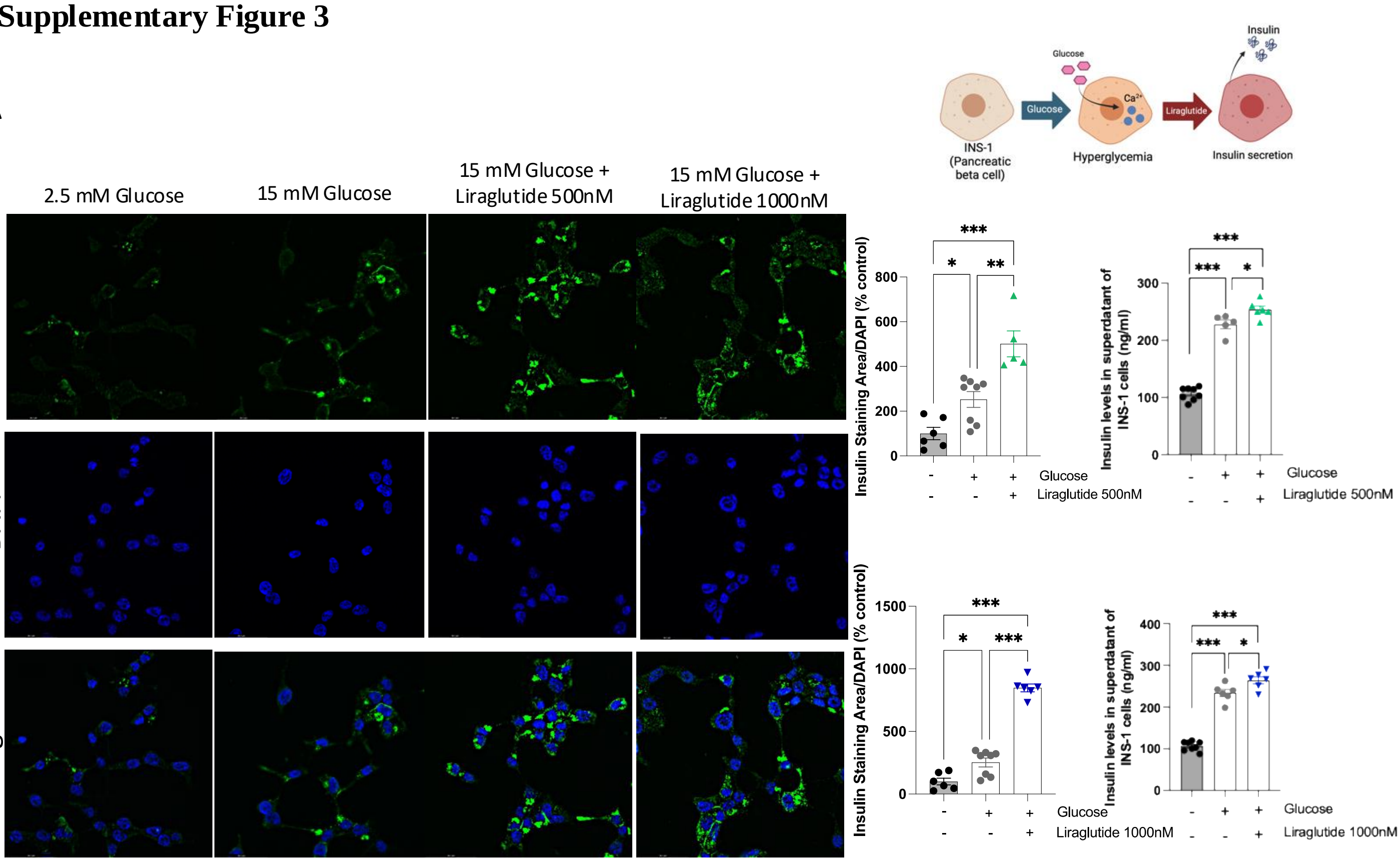

B

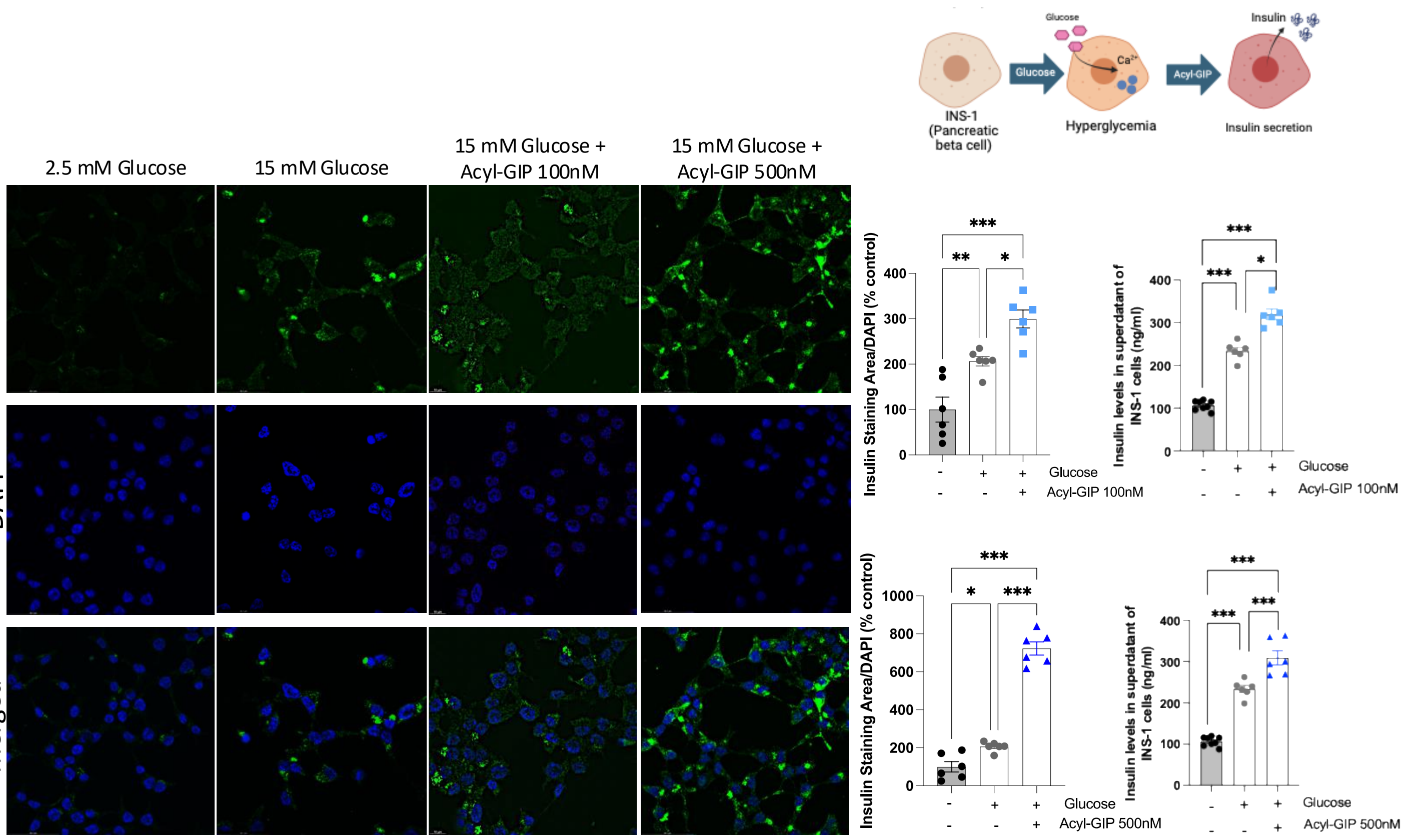

Supplementary Figure 3

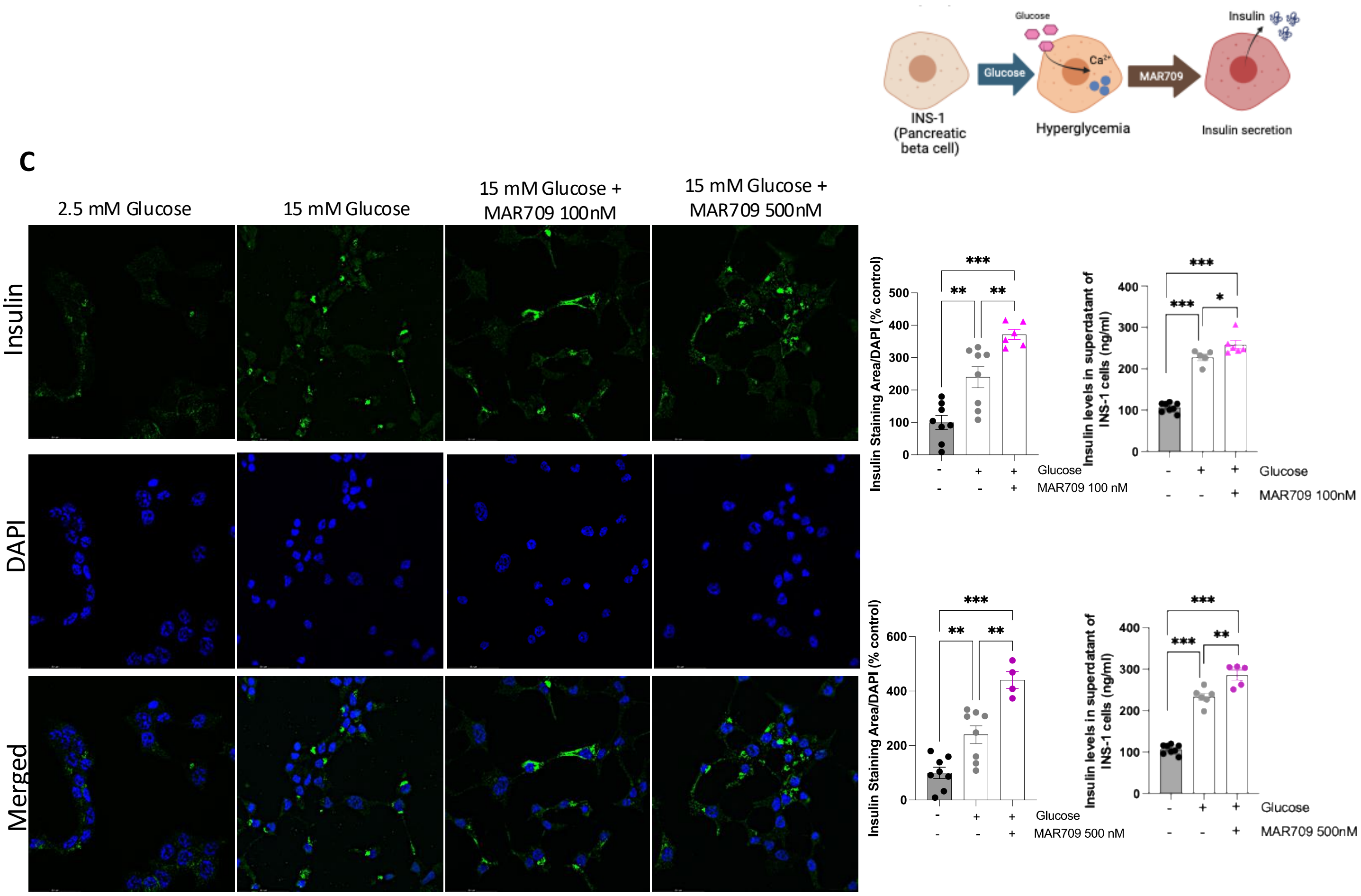

Supplementary Figure 3. Effects of Acyl GIP, Liraglutide and GIP/GLP-1 dual agonist in INS-1 insulinoma cell line. Insulin immunofluorescence representative images, area in INS-1 cells and insulin levels in the medium of INS-1 cells under hyperglycemic conditions treated with (A) Liraglutide (500 and 1000 nM), (B) Acyl-GIP (100 and 500 nM), and (C) MAR709 (100 and 500 nM) (*n*=4-8). Data are presented as mean ± SEM; *p < 0.05, **p < 0.01***, p < 0.001, using a one-way ANOVA followed by a Bonferroni's multiple comparison test.

Supplementary Figure 4

A

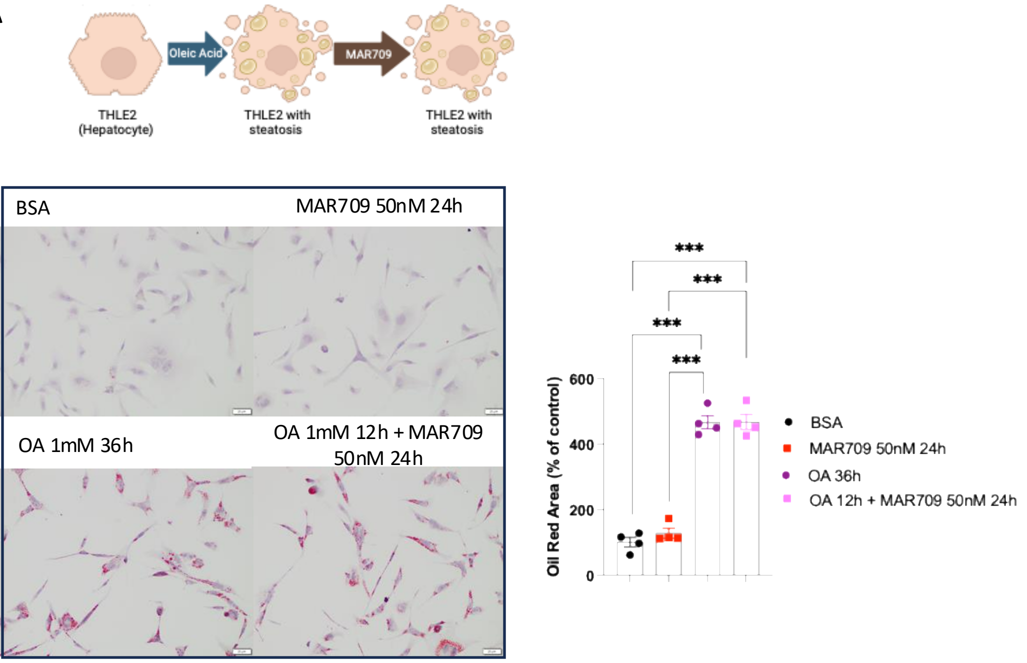

B

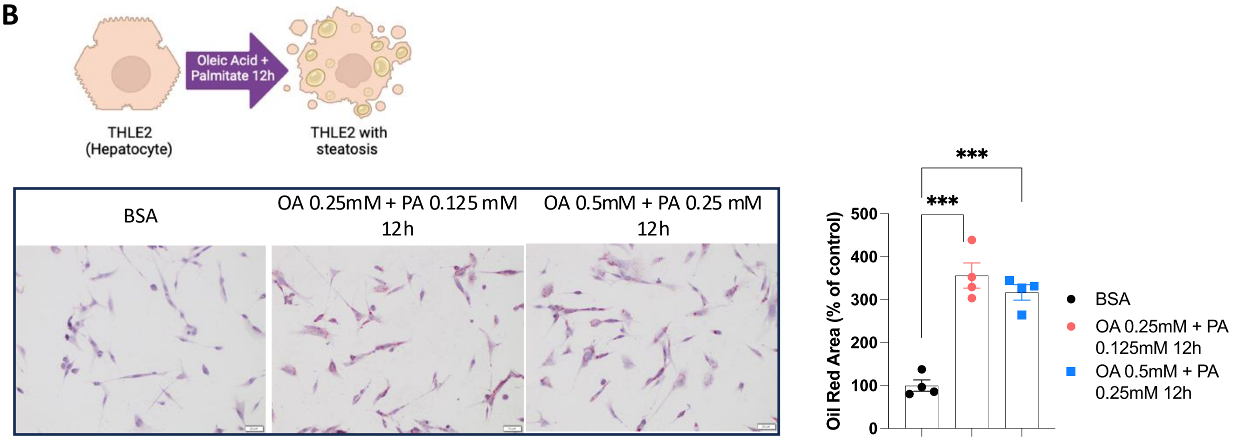

Supplementary Figure 4. Effects of GIP/GLP-1 dual agonist in human hepatocytes cells. (A) Oil red O staining in THLE2 cells treated with or without Oleic Acid (OA) (1mM, 12h) and with GIP/GLP-1 dual agonist (MAR709) (50nM, 24h) (*n*=4). (B) Oil Red O staining in THLE2 cells treated with OA (0.25mM) and PA (0.125mM) and OA (0.5mM) and PA (0.25mM) for 12h (*n*=4). Data are presented as mean ± SEM; ***p < 0.001, using a one-way ANOVA followed by a Bonferroni's multiple comparison test.

Supplementary Figure 5

A

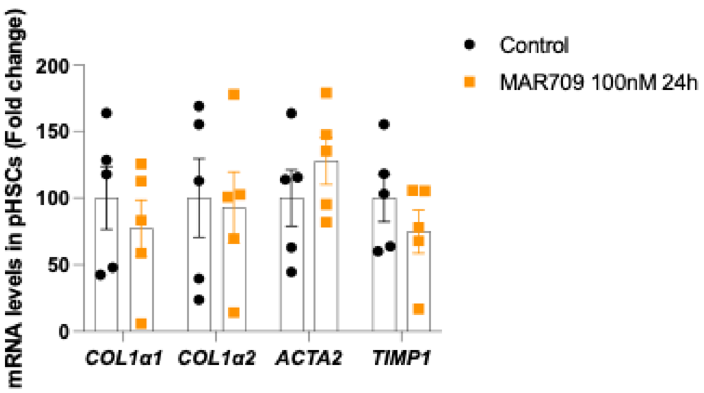

Supplementary Figure 5. Effects of GIP/GLP-1 dual agonist in human primary hepatic stellate cells (pHSCs). (A) mRNA expression of fibrotic markers in human primary hepatic stellate cells (pHSCs) treated with MAR709 (100nM, 24h) (*n*=5). mRNA levels were normalized to the housekeeping gene HPRT. Data are presented as mean ± SEM; **p < 0.01, ***p < 0.001, using a t-test.
