# Supplementary material for: GLP-1 and GIP agonism has no direct actions in human hepatocytes or hepatic stellate cells
Source: Cell Mol Life Sci. 2024 Nov 28;81(1):468. doi: 10.1007/s00018-024-05507-6 (PMC11604888; doi:10.1007/s00018-024-05507-6)

Uncropped Figure 3

Uncropped Figure 3A

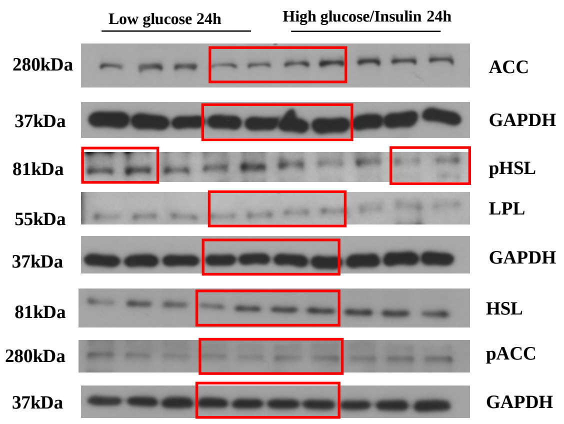

Uncropped Figure 3B

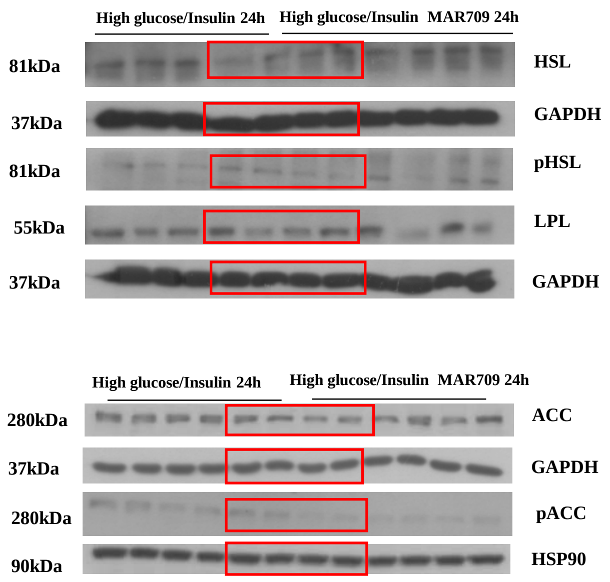

Uncropped Figure 4

Uncropped Figure 4A

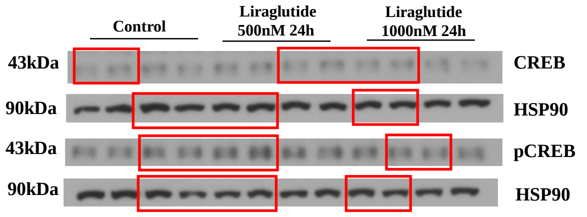

Uncropped Figure 4B

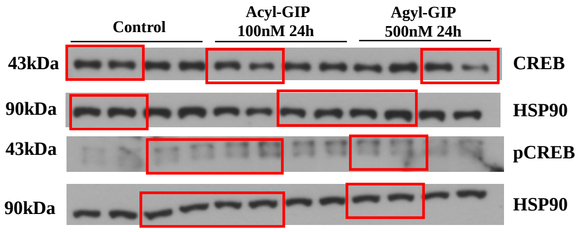

Uncropped Figure 4C

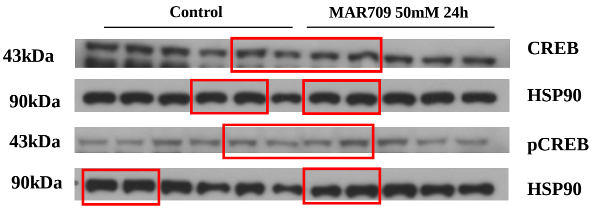

Uncropped Figure 4D

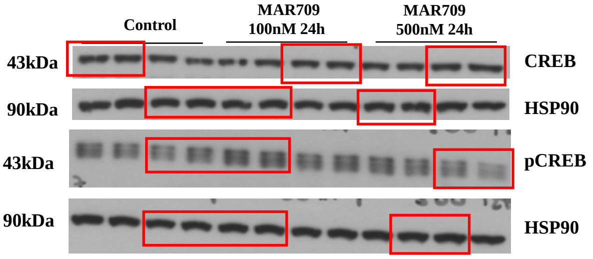

Uncropped Figure 6

Uncropped Figure 6A

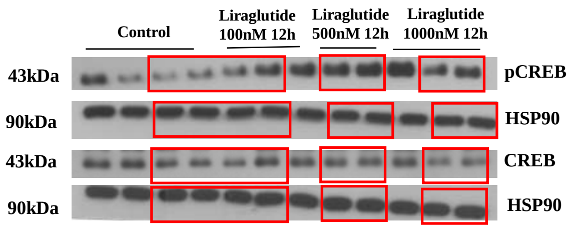

Uncropped Figure 6B

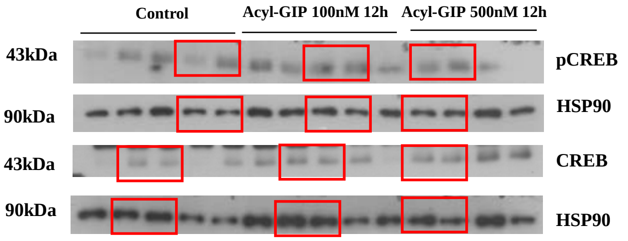

Uncropped Figure 6C

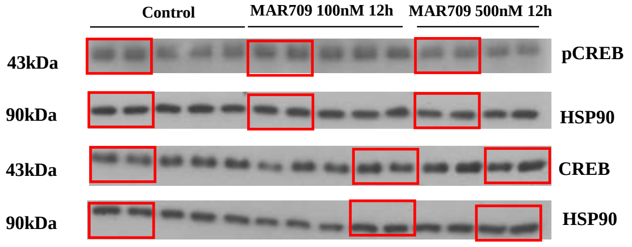

Uncropped Figure 6D

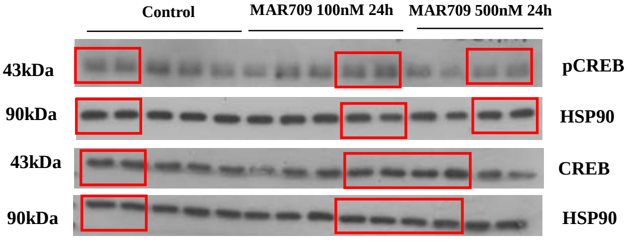

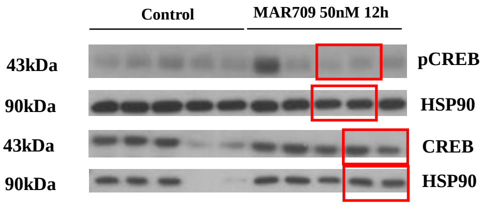

Supplement: Supplementary file 2 — Supplementary file2 (PDF 286 KB) [file 18_2024_5507_MOESM2_ESM.pdf]
